# Unveiling Surface Species Formed on Ni‐Fe Spinel Oxides During the Oxygen Evolution Reaction at the Atomic Scale

**DOI:** 10.1002/advs.202501967

**Published:** 2025-03-31

**Authors:** Weikai Xiang, Sheila Hernandez, Pouya Hosseini, Fan Bai, Ulrich Hagemann, Markus Heidelmann, Tong Li

**Affiliations:** ^1^ Faculty of Mechanical Engineering Atomic‐scale Characterisation Ruhr‐Universität Bochum Universitätsstraße 150 44801 Bochum Germany; ^2^ Faculty of Chemistry and Biochemistry Analytical Chemistry II Ruhr‐Universität Bochum Universitätsstraße 150 44801 Bochum Germany; ^3^ Max‐Planck‐Institut für Nachhaltige Materialien GmbH Max‐Planck‐Straße 1 40237 Düsseldorf Germany; ^4^ Interdisciplinary Center for Analytics on the Nanoscale (ICAN) and Center for Nanointegration Duisburg‐Essen (CENIDE) University of Duisburg‐Essen Carl‐Benz‐Straße 199 47057 Duisburg Germany

**Keywords:** active species, atom probe tomography, OER, oxyhydroxides, Raman spectroscopy, water splitting

## Abstract

Optimizing electrocatalyst performance requires an atomic‐scale understanding of surface state changes and how those changes affect activity and stability during the reaction. This is particularly important for the oxygen evolution reaction (OER) since the electrocatalytically active surfaces undergo substantial reconstruction and transformation. Herein, a multimodal method is employed that combines X‐ray photoemission spectroscopy, transmission electron microscopy, atom probe tomography, operando surface‐enhanced Raman spectroscopy with electrochemical measurements to examine the surface species formed on NiFe_2_O_4_, P‐doped NiFe_2_O_4_ and Ni_1.5_Fe_1.5_O_4_ upon OER cycling. The activated NiFe_2_O_4_ and P‐doped NiFe_2_O_4_ exhibit a significantly lower Tafel slope (≈40 mV dec^−1^) than Ni_1.5_Fe_1.5_O_4_ (≈90 mV dec^−1^), although oxyhydroxides are grown on all three Ni‐Fe spinels during OER. This is likely attributed to the formation of a ≈1 nm highly defective layer with a higher oxygen concentration on the activated NiFe_2_O_4_ and P‐doped NiFe_2_O_4_ nanoparticle surfaces (than that in bulk), which improves the charge transfer kinetics toward OER. Such surface species are not formed on Ni_1.5_Fe_1.5_O_4_. Overall, this study provides a mechanistic understanding of the role of Fe, P, and Ni in forming active oxygen species in the Ni‐based spinels toward OER.

## Graphical abstract

≈1 nm thick amorphous layer with a higher oxygen concentration is formed on the surfaces of pristine P‐doped NiFe_2_O_4_ and activated NiFe_2_O_4_. Such surface layer is thought to significantly promote the charge transfer kinetics compared to the oxyhydroxide layer grown on Ni_1.5_Fe_1.5_O_4_ during the oxygen evolution reaction.

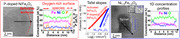

## 1 Introduction

Electrocatalytic water splitting provides a clean solution to produce hydrogen that can be used as chemical fuel in sustainable energy conversion devices.^[^ ^1^ ^]^ However, the targeted efficiency of water electrolysis remains low, mainly due to limitations in electrocatalytic performance at the anode, where the oxygen evolution reaction (OER) occurs.^[^ ^2^ ^]^ Iridium oxides are state‐of‐the‐art OER electrocatalysts due to their optimal combination of activity and long‐term stability toward OER in acidic media.^[^ ^3^ ^]^ However, their scarcity and high costs limit their large industrial energy conversion applications. In this context, earth‐abundant and affordable Ni‐based electrocatalysts, such as hydroxides, oxyhydroxides, metal foams, or oxides, have become cheaper alternatives to noble metal oxide electrocatalysts, since they have outstanding chemical stability and corrosion resistance toward OER, especially in alkaline media.^[^ ^4^ ^]^

Among the Ni‐based OER electrocatalysts, NiFe_2_O_4_ spinel is the least studied and understood since its activity is considerably lower than that of Ni‐based (oxy)hydroxides, iridium, or ruthenium oxides.^[^ ^5^ ^]^ To improve the activity of NiFe‐based spinels, much effort has been devoted to developing new strategies for the optimization of electrocatalysts for OER. For example, doping NiFe_2_O_4_ spinel with non‐metal elements, such as phosphorus (P), possibly improves the adsorption energy of intermediates as well as the electrical conductivity and hydrophilicity,^[^ ^6^ ^]^ thereby enhancing OER activity. However, few studies^[^ ^6^ ^]^ have elucidated the effects of P on the formation of active sites or species in P‐doped NiFe_2_O_4_ spinel toward OER. Alternatively, a decrease in the Fe content of NiFe_2_O_4_ spinel can improve OER activity, as evidenced by the order of OER activity: Ni_1.5_Fe_1.5_O_4_ > NiFe_2_O_4_ > Ni_0.3_Fe_2.7_O_4_.^[^ ^7^ ^]^ However, the effects of Fe on surface reconstruction or transformation of Ni spinels during OER remain elusive.

Essentially, adding small amounts of Fe (≈25 at.%) into Ni oxyhydroxide or hydroxide substantially improves OER activity.^[^ ^8^ ^]^ Although it is generally agreed that oxygen‐oxygen (O─O) bond formation before O_2_ evolution is the rate‐determining step,^[^ ^9^ ^]^ fundamental questions remain highly debated, such as which oxidation state of Fe or Ni is active for OER, and the precise role of Ni and Fe in OER activity.^[^ ^9^, ^10^ ^]^ Nocera and coworkers proposed that Ni(IV) oxo species enhance the OER activity of Fe‐doped Ni oxide films since the Ni(IV) oxo species facilitates O─O bond formation.^[^ ^10c^ ^]^ On the other hand, Stahl and coworkers used operando Mössbauer spectroscopy to provide direct evidence that Fe(IV), formed at the edge, corner, and defect sites of Ni‐Fe layered double hydroxide, is the active site for OER.^[^ ^10a^ ^]^ This evidence was supported by the ab initio theory simulation showing that the formation of Fe(IV) oxo species and the discharge ability of Ni(III) to Ni(II) govern the OER activity of Fe‐doped β‐NiOOH.^[^ ^10d^ ^]^ Additionally, some authors have proposed that negatively charged peroxidic or superoxidic species, possibly NiOO^−^, in Fe‐doped NiOOH are also active for OER.^[^ ^10b^ ^]^ To date, no consensus has been reached for NiFe (oxy)hydroxides. Additionally, few experimental and theoretical studies investigated the effects of Fe and Ni on the formation of surface species of NiFe spinels toward OER.^[^ ^11^ ^]^ Systematic work is needed to clarify how Fe and Ni affect the OER activity of the Ni‐Fe spinels. Knowledge derived from Ni oxyhydroxide or hydroxide might not be transferred to Ni spinels, as they have completely different structures.

The conflicting literature findings possibly originate from the fact that OER electrocatalyst surfaces undergo dynamic and severe surface reconstruction and transformation. For example, Co‐based spinel surfaces have been found to transform into a few nanometres (nm) thick X‐ray amorphous Co oxyhydroxide layer that serves as active species toward OER.^[^ ^12^ ^]^ In order to reveal the activation or deactivation mechanisms of electrocatalysts, we must first know the valence state, morphology, and composition of electrocatalyst surfaces at different stages of the reactions, and thus correlate their dynamic changes with activity and stability. Although operando and in situ, spectroscopy techniques have provided a wealth of valuable information regarding the valence state, it is nearly impossible to obtain all the necessary information regarding the surface state using a single experimental technique.^[^ ^10^, ^13^ ^]^ Additionally, acquiring atomic‐scale structural and compositional details regarding the topmost atomic layers of electrocatalysts remains challenging for most techniques. This lack of experimental information has hindered a complete evaluation of the contribution made by individual atoms in this extended zone to the interplay between catalytic activity, selectivity, and stability. Therefore, in order to elucidate active species or sites, it is essential to establish a multimodal method that can link, the valence state, structure, morphology, and elemental distribution of electrocatalyst surfaces at the atomic scale with activity and stability during electrocatalytic reactions.

Atom probe tomography (APT), which can be used to identify and map the distribution of individual elements of materials with sub‐nanometer spatial resolution in 3D, is well‐suited to study the surface composition changes of (electro)catalysts.^[^ ^14^ ^]^ Herein, we combine X‐ray photoemission spectroscopy (XPS), transmission electron microscopy (TEM), APT, and operando surface‐enhanced Raman spectroscopy (SERS), with electrochemical measurements to investigate the changes in oxidation state, structure, composition, and elemental distribution of NiFe_2_O_4_, P‐doped NiFe_2_O_4,_ and Ni_1.5_Fe_1.5_O_4_ spinel nanoparticles in alkaline media under OER conditions. The aims of this study are to: (i) elucidate the chemical nature of surface species, and (ii) clarify the role of P and Fe for the OER activity of Ni‐based spinel‐type oxides. We reveal the formation of ≈1 nm thick amorphous layer with a higher oxygen concentration on the surfaces of pristine P‐doped NiFe_2_O_4_ and activated NiFe_2_O_4_. Such surface layer is thought to significantly promote the OER charge transfer kinetics compared to the oxyhydroxides grown on Ni_1.5_Fe_1.5_O_4_ during OER.

## 2 Results and Discussion

**Figure 1:**
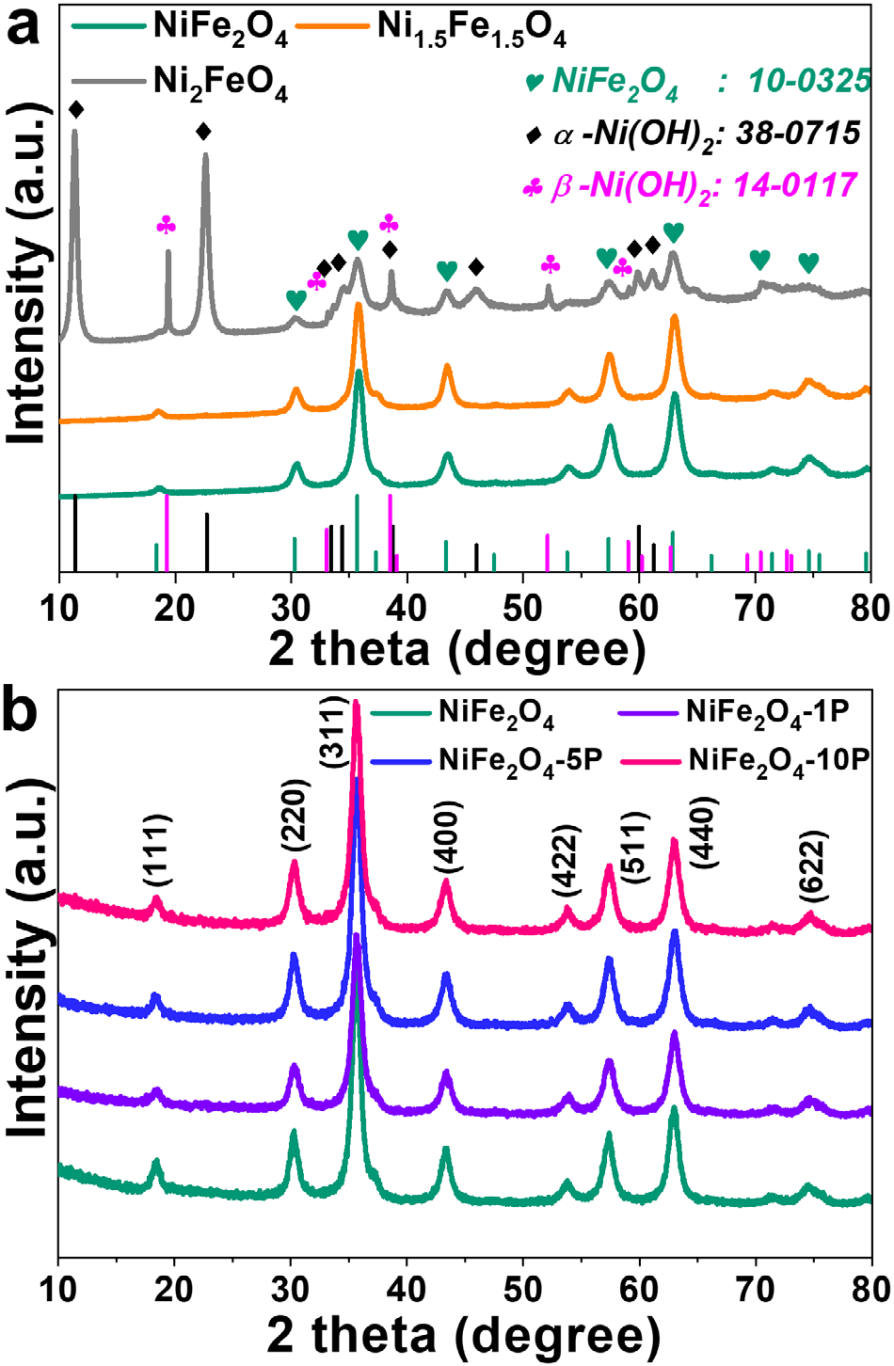
Powder X‐ray diffraction patterns of a,b) pristine NiFe_2_O_4_, Ni_1.5_Fe_1.5_O_4_, Ni_2_FeO_4_, NiFe_2_O_4_‐1P, NiFe_2_O_4_‐5P, and NiFe_2_O_4_‐10P nanoparticles.

Ni‐Fe spinel oxide nanoparticles with varying Ni/Fe ratios (1:1, 1:2, and 2:1) were synthesized by the hydrothermal method (see Supporting Information). P‐doped NiFe_2_O_4_ nanoparticles were prepared via the calcination of NiFe_2_O_4_ and NaH_2_PO_2_·H_2_O in N_2_ at 300 °C; varying P contents were doped by changing the amounts of NaH_2_PO_2_·H_2_O, yielding the oxide stoichiometries of Ni_0.9_Fe_2.1_P_0.1_O_4_, Ni_0.8_Fe_2.0_P_0.2_O_4_ and Ni_0.8_Fe_1.9_P_0.3_O_4_ (measured by ICP‐MS, see Table S1, Supporting Information), termed NiFe_2_O_4_‐1P, NiFe_2_O_4_‐5P and NiFe_2_O_4_‐10P. **Figure** **1**a,b depicts the X‐ray diffraction (XRD) data of all the pristine nanoparticles. The NiFe_2_O_4_ and Ni_1.5_Fe_1.5_O_4_ samples exhibit a cubic spinel structure (Fd $\bar{3}$ m) ^[^ ^15^ ^]^ with a lattice constant of 8.335 Å, whereas the Ni_2_FeO_4_ sample contains α‐ and β‐Ni(OH)_2_^[^ ^16^ ^]^ in addition to the cubic spinel phase (Figure 1a). This result suggests that the cubic spinel structure becomes unstable by forming α‐ and β‐Ni(OH)_2_ when the Ni/Fe ratio exceeds 1:1. For P‐doped NiFe_2_O_4_, all pristine systems have the cubic spinel structure without any secondary phases (e.g., iron phosphate) or lattice parameter changes (Figure 1b). TEM reveals that the Ni‐Fe mixed spinel and P‐doped NiFe_2_O_4_ nanoparticles have a spherical morphology with a diameter of ≈10 nm (Figure S1, Supporting Information).

**Figure 2:**
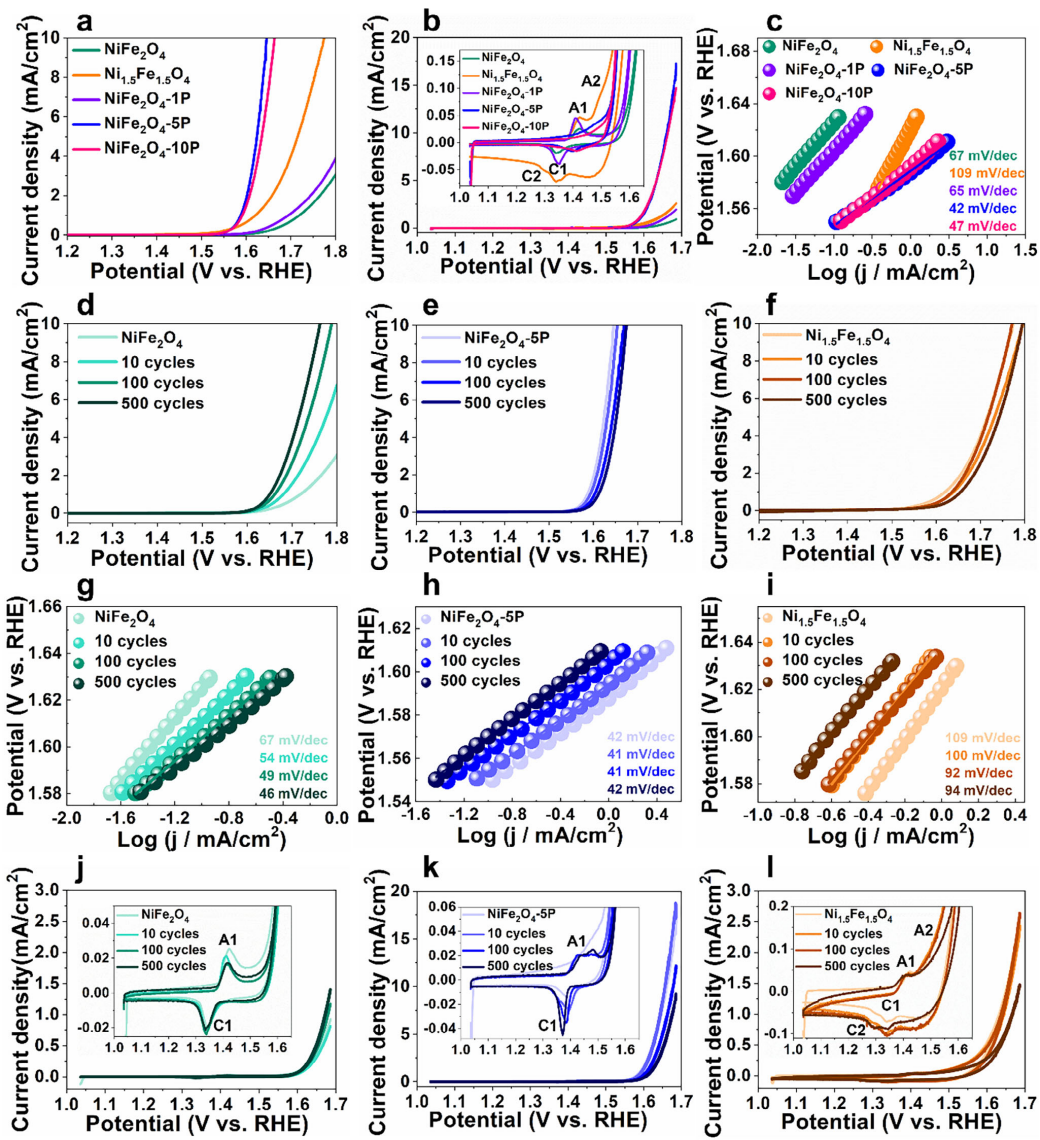
a) Linear sweep voltammetry (LSV), b) cyclic voltammetry (CV) curves, c) Tafel slopes of pristine Ni_1.5_Fe_1.5_O_4_, NiFe_2_O_4_, NiFe_2_O_4_‐1P, NiFe_2_O_4_‐5P, and NiFe_2_O_4_‐10P nanoparticles. d–f) LSV curves, g–i) Tafel slopes, and j–l) CV curves of NiFe_2_O_4_, NiFe_2_O_4_‐5P, and Ni_1.5_Fe_1.5_O_4_ nanoparticles in the pristine state and after 10, 100, and 500 CV cycles. The electrochemical data were recorded in oxygen‐saturated 1.0 m purified KOH with 95% iR compensation. Tafel slopes were calculated from the corresponding LSV curves.

To compare the OER activity, we performed linear sweep voltammetry (LSV) and cyclic voltammetry (CV) on pristine NiFe_2_O_4_, Ni_1.5_Fe_1.5_O_4,_ and P‐doped NiFe_2_O_4_ nanoparticles using a rotating disk electrode in oxygen‐saturated 1.0 m purified KOH^[^ ^17^ ^]^ under OER conditions. The current density was normalized by the geometric surface area of the glassy carbon electrode (0.196 cm^2^). LSV curves displaying current densities normalized to the estimated effective electrochemical active surface area (ECSA), are shown in Figure S2g–i (Supporting Information). Ohmic drop (*iR*) correction (95%) was applied to compensate for the potential loss resulting at the electrode as compared to the nominally applied potential due to current flux in the highly resistive system.^[^ ^18^ ^]^ Given that the α‐ and β‐Ni(OH)_2_ in Ni_2_FeO_4_ could contribute different active sites or reaction mechanisms from NiFe_2_O_4_ and Ni_1.5_Fe_1.5_O_4_ spinels, Ni_2_FeO_4_ was excluded from the electrochemical performance comparison. The LSV curves, shown in **Figure** **2**a, demonstrate that NiFe_2_O_4_‐5P (i.e., Ni_0.8_Fe_2.0_P_0.2_O_4_ with P concentration of ≈3 at.%) exhibits the highest OER activity (dark blue curve), as its overpotential (≈415 mV measured at 10 mA cm^−2^) is remarkably lower than that of pristine NiFe_2_O_4_ (> 600 mV, green curve). The OER activity of Ni_1.5_Fe_1.5_O_4_ (with an overpotential of ≈548 mV at 10 mA cm^−2^) is slightly higher than that of NiFe_2_O_4_ in agreement with a previous study^[^ ^7^ ^]^ that found that lowering the Fe/Ni in Ni‐Fe mixed spinel improves OER activity.

A similar trend was seen in the CV curves of pristine spinels after the first cycle, in Figure 2b, where the OER current densities at 1.69 V versus RHE decrease in the following order: NiFe_2_O_4_‐5P > NiFe_2_O_4_‐10P ≫ Ni_1.5_Fe_1.5_O_4_ > NiFe_2_O_4_‐1P > NiFe_2_O_4_. The Tafel slopes, depicted in Figure 2c and derived from the LSV curves of Figure 2a, show that adding a small amount of P (≈1.4 at.% for NiFe_2_O_4_‐1P) yields a similar Tafel slope (≈65 mV dec^−1^) to that of NiFe_2_O_4_ (≈67 mV dec^−1^). As P doping increases to ≈3 at.%, the charge transfer kinetics of NiFe_2_O_4_‐5P is significantly enhanced (≈42 mV/dec). This finding is consistent with a previous study that found that P‐doped NiFe_2_O_4_ nanosheets grown on carbon cloth exhibit a low Tafel slope (49 mV dec^−1^) and overpotential (231 mV at 10 mA cm^−2^).^[^ ^6a^ ^]^ Note that the large discrepancies in the overpotential between their work^[^ ^6a^ ^]^ and our study (≈415 mV at 10 mA cm^−2^) are because the ECSA of the P‐doped NiFe_2_O_4_ nanosheets is significantly higher than our nanoparticles. When normalizing the current densities to the respective ECSAs,^[^ ^6a^ ^]^ the specific current density of the NiFe₂O₄‐5P nanoparticles in our study is ≈10 times higher than that of the P‐doped NiFe_2_O_4_ nanosheets on carbon cloth.^[^ ^6a^ ^]^ Thus, the NiFe₂O₄‐5P prepared in this study possesses excellent intrinsic electrocatalytic activity for OER. Additionally, the Tafel slope of Ni_1.5_Fe_1.5_O_4_ is the highest (≈109 mV dec^−1^), suggesting that Ni_1.5_Fe_1.5_O_4_ possibly exhibits different surface species compared to those of NiFe_2_O_4_ and P‐doped NiFe_2_O_4_.

To examine the activity changes during OER cycling, we performed LSV on NiFe_2_O_4_‐5P (the most active), NiFe_2_O_4_ (the least active), and Ni_1.5_Fe_1.5_O_4_ after different numbers of CV cycles. Figure 2d–i shows their respective LSV curves and derived Tafel slopes in the pristine state and after 10, 100, and 500 CV cycles. Interestingly, NiFe_2_O_4_ undergoes a remarkable activation process, as indicated by considerable decreases in overpotentials with an increasing number of CV cycles (Figure 2d,g). Specifically, the overpotential of NiFe_2_O_4_ decreases to ≈570 mV (at 10 mA cm^−2^) after 500 CV cycles. This activation might be associated with increased ECSA after OER cycling (see ECSA of post‐OER samples in Figure S2j, Supporting Information). On the other hand, the Tafel slope of NiFe_2_O_4_ decreases from ≈67 mV dec^−1^ in the pristine state to ≈46 mV dec^−1^ after 500 CV cycles (Figure 2g), reaching a similar Tafel slope as the pristine NiFe_2_O_4_‐5P (≈42 mV dec^−1^). This result indicates that active species or sites are possibly generated on NiFe_2_O_4_ during OER cycling, aiding a more rapid charge transfer. In comparison, the overpotential of NiFe_2_O_4_‐5P increases slightly, by ≈30 mV after 500 CV cycles (Figure 2e–h), accompanied by almost no changes to the Tafel slopes (Figure 2h,i), implying that a slight deactivation of NiFe_2_O_4_‐5P upon OER cycling. Notably, although the Tafel slope of activated NiFe_2_O_4_ drops upon OER cycling, the overpotential of activated NiFe_2_O_4_ (≈570 mV at 10 mA cm^−2^) is still higher than that of NiFe_2_O_4_‐5P after 500 CV cycles (≈445 mV at 10 mA cm^−2^), Figure 2d,e. This might be attributed to the fact that the ECSA of activated NiFe_2_O_4_ is lower than that of P‐doped NiFe_2_O_4_ upon OER cycling (see Figure S2j,l, Supporting Information). For Ni_1.5_Fe_1.5_O_4_, the corresponding overpotential increases slightly but the Tafel slope decreases from ≈109 mV dec^−1^ in the pristine state to ≈94 mV dec^−1^ after 500 cycles, inferring the formation of active species that promote OER kinetics. Alternatively, the coverage of hydroxide ions might increase, leading to a decrease in the Tafel slope.

Notably, NiFe_2_O_4_, NiFe_2_O_4_‐5P and Ni_1.5_Fe_1.5_O_4_ exhibit distinctly different redox peaks during OER cycling, as revealed by CV curves in the insets of Figure 2b,j–l. A pair of redox peaks (A1/C1) at ≈1.42 and ≈1.34 V versus RHE was observed for NiFe_2_O_4_ (Figure 2j, insets), which is attributed to the Ni(II)↔Ni(III) transition often observed in Ni‐based oxides and hydroxides during OER.^[^ ^19^ ^]^ As the number of CV cycles increases, the anodic A1 peak of NiFe_2_O_4_ shifts to lower potentials, further confirming the activation of NiFe_2_O_4_ upon OER cycling.^[^ ^20^ ^]^ In comparison, the cathodic C1 peak of pristine NiFe_2_O_4_‐5P occurs at ≈1.40 V versus RHE, close to the anodic A1 peak (Figure 2k, insets). As OER proceeds, the C1 peak becomes more pronounced and shifts to a lower potential of ≈1.37 V versus RHE accompanied by a shift of A1 to a higher potential of ≈1.48 V versus RHE (Figure 2k), implying a gradual deactivation of NiFe_2_O_4_‐5P with increasing CV cycles. For pristine Ni_1.5_Fe_1.5_O_4_, the CV curves exhibit the C1 peak at ≈1.34 V versus RHE and a second cathodic peak (C2) at ≈1.29 V versus RHE (Figure 2l, insets), which become evident after 500 CV cycles. The additional C2 peak might be induced due to a small amount of α‐ and β‐Ni(OH)_2_ impurities in the Ni_1.5_Fe_1.5_O_4_ nanoparticle sample, despite their absence from the XRD data (Figure 1a). These hydroxides likely contribute to the high overpotential of Ni_1.5_Fe_1.5_O_4._^[^ ^21^ ^]^ This explains its poor OER activity, as decreasing the Fe content in NiFe_2_O_4_ spinel was thought to improve the OER activity according to previous work.^[^ ^7^ ^]^ The C2 peak most likely corresponds to the Ni(OH)_2_ hydroxide ↔ NiOOH oxyhydroxides transition, as reported in the previous literature.^[^ ^22^ ^]^ The gradual transformation to oxyhydroxides might improve the charge transfer kinetics of Ni_1.5_Fe_1.5_O_4_, as indicated by Tafel slopes of Ni_1.5_Fe_1.5_O_4_ in Figure 2i. In summary, P doping substantially enhances the OER activity of NiFe_2_O_4_. The pristine NiFe_2_O_4_ is activated upon OER cycling, reaching similar low Tafel slopes (≈46 mV dec^−1^) as pristine NiFe_2_O_4_‐5P (≈42 mV dec^−1^). In comparison, Ni_1.5_Fe_1.5_O_4_ is slightly activated after OER, albeit with high Tafel slopes (≈94 mV dec^−1^). The redox peaks of NiFe_2_O_4_ and NiFe_2_O_4_‐5P are more pronounced than those of Ni_1.5_Fe_1.5_O_4_ (Figure 2j–l), implying that the redox reaction of NiFe_2_O_4_ and NiFe_2_O_4_‐5P might be different from that of Ni_1.5_Fe_1.5_O_4_. One might speculate that the pronounced redox peaks for NiFe_2_O_4_ and NiFe_2_O_4_‐5P arise from α‐Ni(OH)₂ ↔ γ–NiOOH, while less significant redox peaks in CV curves of Ni_1.5_Fe_1.5_O_4_ might correspond to β‐Ni(OH)_2_ ↔ β‐NiOOH based on the redox peak intensity difference of α‐Ni(OH)₂/γ–NiOOH and β‐Ni(OH)_2_/β‐NiOOH reported in the previous work.^[^ ^23^ ^]^ Such surface transformation will be investigated and discussed in the following section.

**Figure 3:**
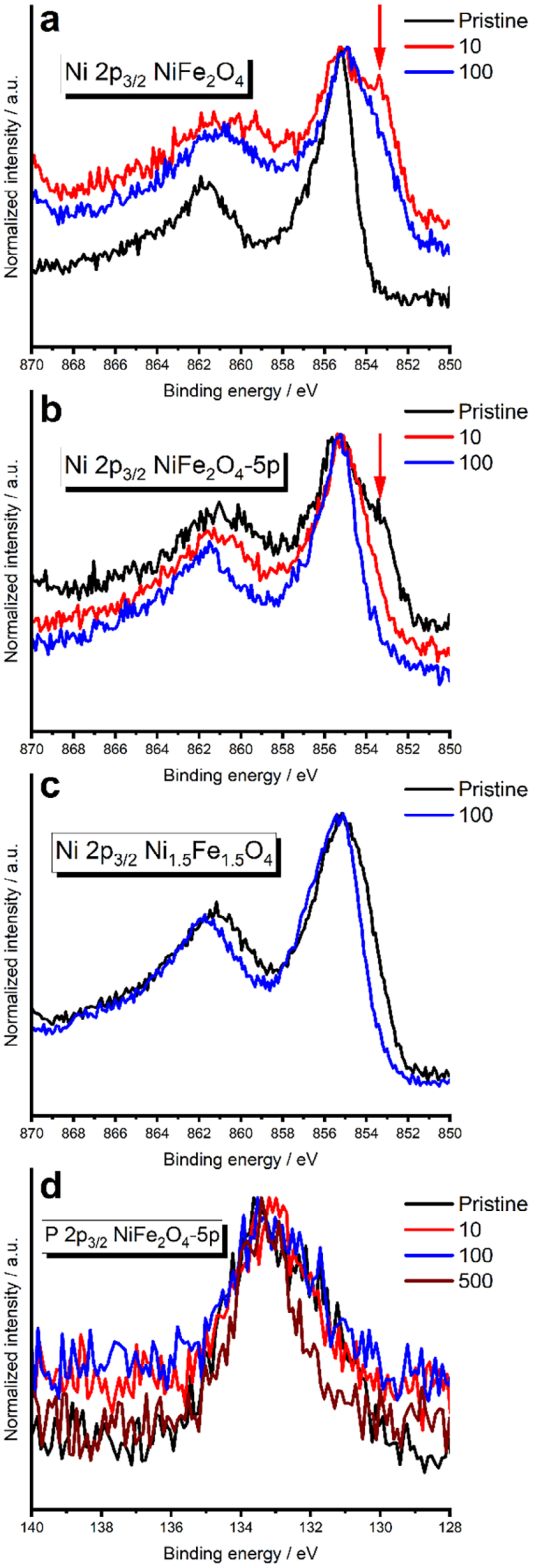
Evolution of oxidation state. a–c) the normalized Ni 2p_3/2_ spectra of NiFe_2_O_4_, NiFe_2_O_4_‐5P, and Ni_1.5_Fe_1.5_O_4_ nanoparticles at the pristine state and after 10 and 100 cycles and d) P 2p_3/2_ spectra of NiFe_2_O_4_‐5P nanoparticles in the pristine state as well as after 10, 100 and 500 CV cycles of OER.

To elucidate the causes of NiFe_2_O_4_ activation and clarify the roles of P and Fe in the improved OER activity of NiFe_2_O_4_, we first performed XPS on NiFe_2_O_4_, NiFe_2_O_4_‐5P, and Ni_1.5_Fe_1.5_O_4_ in the pristine state and after 10 and 100 CV cycles. The Ni 2p and O 1s spectra of pristine NiFe_2_O_4_, NiFe_2_O_4_‐5P, and Ni_1.5_Fe_1.5_O_4_ are shown in **Figures** **3**a–c and S3 (Supporting Information). Given that the Ni 2p_3/2_ peaks of Ni(III) and Ni(II) from NiOOH, NiFe_2_O_4_ and Ni(OH)_2_ overlap at ≈854.6‐855.7 eV,^[^ ^24^ ^]^ it is difficult to distinguish Ni(III) and Ni(II) from the main peak at ≈855 eV. Thus, Ni 2p_3/2_ spectra were analyzed by normalizing the maximum intensity to reveal changes before and after OER. Interestingly, in addition to the main peak at ≈855 eV, another peak at ≈853 eV was observed in the Ni 2p_3/2_ spectra of NiFe_2_O_4_ after 10 CV cycles (red curve, Figure 3a), as pointed by the red‐colored arrow. Although the additional peak disappears after 100 CV cycles (blue curve, Figure 3a), the intensity of the spectra in the region of ≈853 eV is still higher than that of the pristine state of NiFe_2_O_4_ (black curve, Figure 3a). Such an unusual peak was also observed for Ni 2p_3/2_ spectra of pristine NiFe_2_O_4_‐5P (black curve, Figure 3b). The additional peak in Ni 2p_3/2_ spectra at lower binding energy suggests the presence of lower oxidation state Ni for post‐OER NiFe_2_O_4_ and pristine NiFe_2_O_4_‐5P, since this peak is located in between that of NiO(II) and Ni(0) metal.^[^ ^24a^ ^]^ By relating the electrochemical data (Figure 2b,g,k), we speculate that the activation of NiFe_2_O_4_ and the high activity of pristine NiFe_2_O_4_‐5P are most likely correlated with the presence of lower oxidation state Ni. Additionally, the O 1s spectra of post‐OER NiFe_2_O_4_ (after 10 and 100 CV cycles) and pristine NiFe_2_O_4_‐5P, shown in Figure S3b–d, Supporting Information) show a peak with a relatively lower binding energy of ≈528.4 eV, termed 528.4 eV‐O, which was not observed for other samples or electrochemical conditions (Figure S3, Supporting Information). Notably, shifts of XPS spectra might also indicate the changes in the local atom environment since such lower oxidation state Ni has also been detected by XPS in highly defective NiO,^[^ ^25^ ^]^ wherein the shifts of O 1s and Ni 2p spectra are caused by adsorption of oxygen‐containing species at the defects in NiO. This suggests that certain highly defective surface species are created in activated NiFe_2_O_4_ and pristine NiFe_2_O_4_‐5P, contributing to the improved charge transfer kinetics during OER.

In contrast, no additional Ni 2p_3/2_ peak (at ≈853 eV) and 528.4eV‐O peak were observed for Ni_1.5_Fe_1.5_O_4_ in the pristine state or after 100 cycles (Figure 3c and Figure S3g,h, Supporting Information). Instead, the surface of Ni_1.5_Fe_1.5_O_4_ is covered by hydroxyl ions after 100 cycles of OER, likely forming oxyhydroxides, as indicated by the pronounced O2 peak at 531.0 eV (Figure S3 h, Supporting Information, orange curve); this O2 peak often suggests the presence of chemisorbed oxygen on the surface, such as hydroxyl‐like groups.^[^ ^26^ ^]^ Although partial charging may shift the O 1s peak to lower binding energies, the consistent observations across multiple samples and agreement with electrochemical data strongly support the presence of lower oxidation state Ni or defect‐rich surfaces rather than an artifact. Note that XPS data of the Fe 2p region is not shown, as it exhibits a significant overlap with the Ni LMM Auger peak. Due to the utilization of an Al *K*α X‐ray source, this overlap rendered the Fe 2p region unsuitable for in‐depth analysis.^[^ ^27^ ^]^

P doping in NiFe_2_O_4_ introduces complexity to the electronic structure and surface properties, as evidenced by the P 2p spectra shown in Figure 3d. Initially, the P 2p spectrum (Figure 3d, black curve) exhibits two distinct peaks: one at a lower binding energy of ≈131.4 eV and another at ≈133.4 eV (see Figure S4, Supporting Information). The lower binding energy peak (at 131.4 eV) is typically associated with metal phosphides or P in a more reduced state, suggesting a strong interaction between the dopant and the host lattice.^[^ ^28^ ^]^ The higher binding energy peak at 133.4 eV is characteristic of phosphate species, indicating the presence of more oxidized phosphorus environments. Intriguingly, after a long OER duration (500 cycles), the lower binding energy peak at 131.4 eV disappears, while the higher binding energy peak (133.4 eV) persists (Figure 3d, green curve, and Figure S4, Supporting Information). The disappearance of the 131.4 eV peak suggests that the more reduced phosphorus species might leach out or be oxidized and incorporated into NiFe_2_O_4_ during OER. By relating O 1*s* and Ni 2p spectra of pristine NiFe_2_O_4_‐5P, we speculate that reduced state P likely contributes to active species formation, while P possibly dissolves or transforms, likely in the form of phosphate species according to the Pourbaix diagram,^[^ ^29^ ^]^ as OER proceeds.

**Figure 4:**
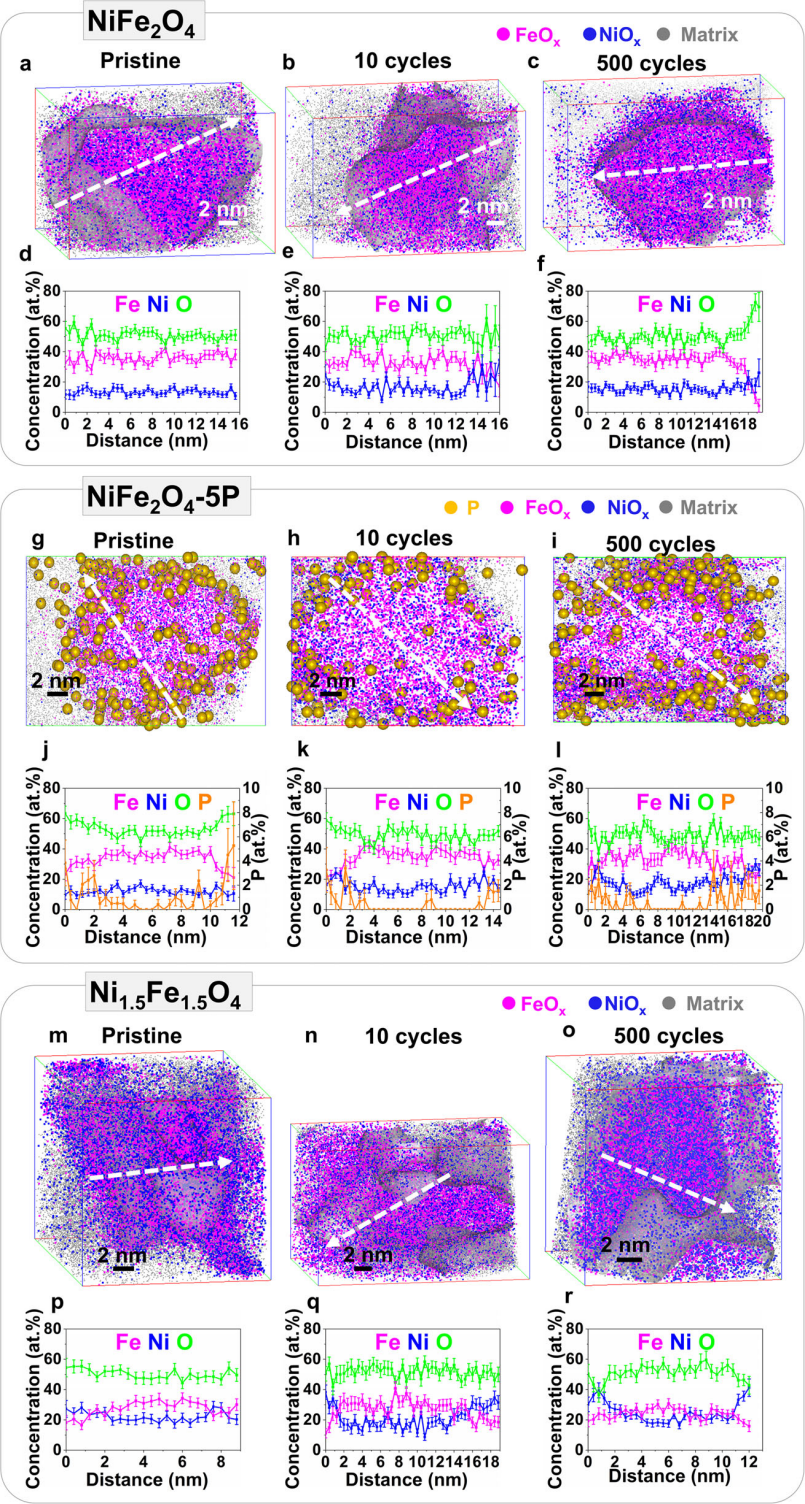
3D atom maps of representative NiFe_2_O_4_ nanoparticles a) in the pristine state and after b) 10 and c) 500 CV cycles, and d–f) 1D concentration profiles plotted along the arrow directions in (a–c), cross‐sectional atom maps of NiFe_2_O_4_‐5P nanoparticles g) in the pristine state and after h) 10 and i) 500 CV cycles, and j–l) 1D concentration profiles measured along the arrow directions in (g–i). 3D atom maps of representative Ni_1.5_Fe_1.5_O_4_ nanoparticles m) in the pristine state and after n) 10 and o) 500 CV cycles and p–r) 1D concentration profiles plotted along the arrow directions in (m–o). Additional APT analyses were provided in Figures S5–S7 (Supporting Information).

**Figure 5:**
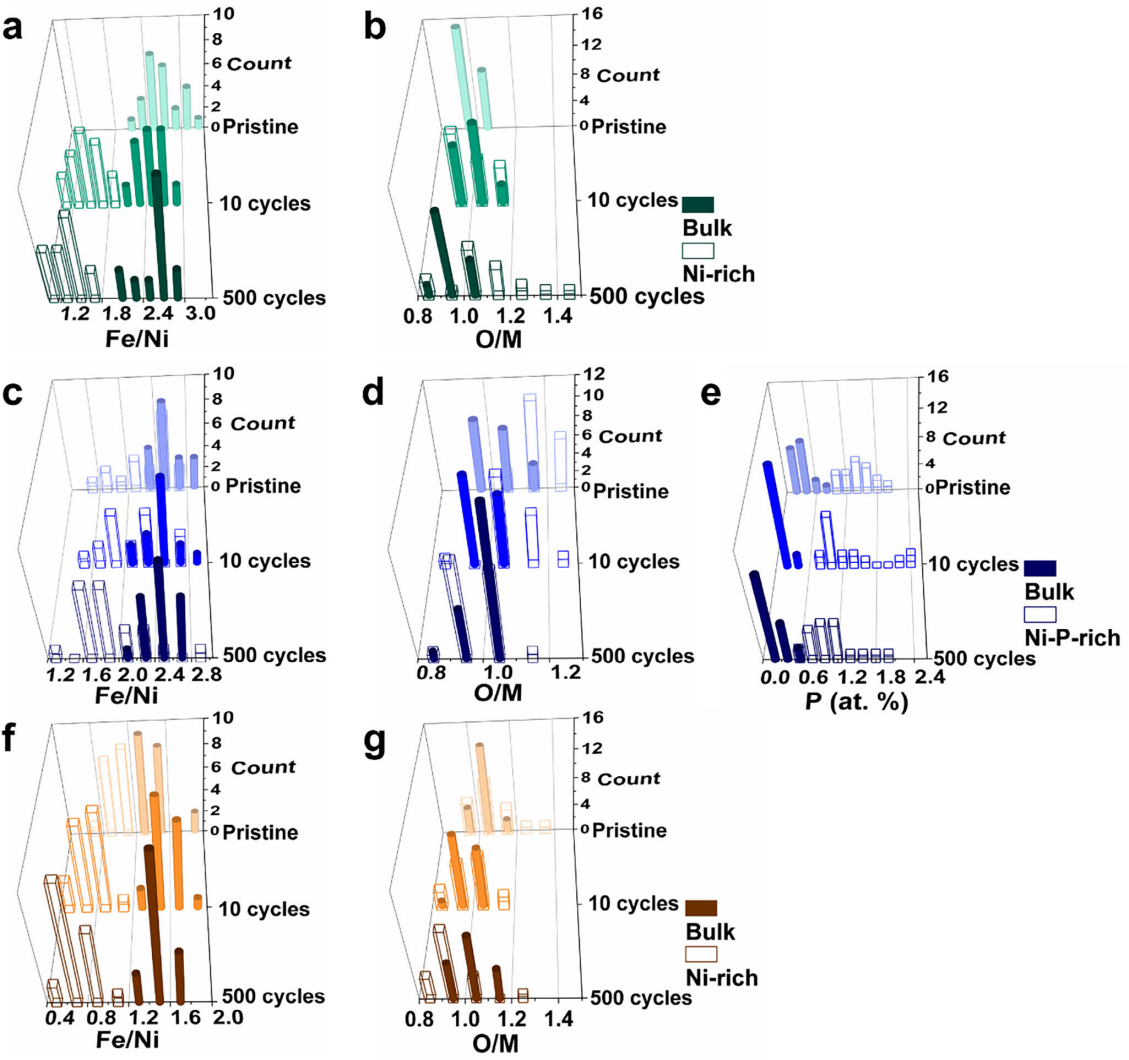
Compositional histograms of a) Fe/Ni ratio and b) O/M ratio (M = Ni + Fe) in the bulk‐ and Ni‐rich regions of NiFe_2_O_4_ nanoparticles, compositional histograms of c) Fe/Ni ratio, d) O/M ratio, and e) P concentrations in the bulk‐ and Ni‐P‐rich regions of NiFe_2_O_4_‐5P nanoparticles, and compositional histograms of f) Fe/Ni ratio and g) O/M ratio in the Ni‐rich and bulk regions of Ni_1.5_Fe_1.5_O_4_ nanoparticles in the pristine state and after 10 and 500 CV cycles.

To investigate the species formed at NiFe_2_O_4_ and NiFe_2_O_4_‐5P nanoparticle surfaces, we employed APT and TEM to examine their structural and compositional changes before and after OER and compare them with Ni_1.5_Fe_1.5_O_4_. APT relies on the field evaporation of surface atoms from a needle‐like specimen, during which the sub‐nanometer spatial coordinates and chemical identity are defined by a single‐ion position‐sensitive detector and a built‐in time‐of‐flight mass spectrometry, respectively.^[^ ^30^ ^]^ APT data take the form of 3D point clouds, where every point represents an individual ion detected and elementally identified. **Figure** **4**a–c shows the 3D‐APT reconstruction of representative NiFe_2_O_4_ nanoparticles in their pristine state and after 10 and 500 CV cycles, respectively, where blue and magenta dots indicate the Fe‐ (FeO_x_) and Ni‐containing (NiO_x_) molecular ions (additional APT data are shown in Figure S5, Supporting Information). Note that NiO_x_ and FeO_x_ are molecular ions decomposed from NiFe spinel oxides during APT measurements. The surface compositions of the nanoparticles were resolved by plotting 1D concentration profiles along the arrows in Figure 4a–c (Figure 4d–f). The averaged surface compositions and associated compositional histograms obtained from all nanoparticles, both in their pristine state and after OER, are summarized in **Table** **1** and **Figure** **5**a,b. Figure 4d reveals that Ni and Fe are distributed uniformly across the pristine NiFe_2_O_4_ nanoparticle, with a Fe/Ni ratio of ≈2.6 (Table 1); this yields an oxide stoichiometry of Ni_0.9_Fe_2.1_O_4_. After 10 CV cycles, the surface of the NiFe_2_O_4_ nanoparticle is enriched with Ni, yielding a decreased Fe/Ni ratio of ≈1.5 (Table 1, Figures 4e and 5a). As OER proceeds, the oxygen concentration in the Ni‐rich surfaces of the NiFe_2_O_4_ nanoparticles increases, as evidenced by the oxygen/total metal cation (O/M) ratio approaching 1.2–1.4 after 500 cycles (Figures 5b and 4f), significantly higher than that in nanoparticle bulk (≈1.0). Note that the O/M ratio of pristine NiFe_2_O_4_ detected by APT (≈1.1) is lower than the theoretical spinel stoichiometry of ≈1.3. A similarly low oxygen concentration was detected by APT for mixed CoFe spinel nanoparticles,^[^ ^31^ ^]^ where the oxygen deficiency was attributed to multiple hits, e.g.,^16^O_2_^2+^ at 16 Da in mass spectra, leading to an underestimated oxygen content. Despite this underestimation, changes in oxygen content after various numbers of OER cycles can still be used to evaluate surface changes following OER. The compositional histograms and averaged compositions (Figure 5a,b and Table 1) indicate that Fe dissolves, possibly in the formation of soluble FeO_4_^2‐^ according to Fe Pourbaix diagram,^[^ ^32^ ^]^ from the surfaces of NiFe_2_O_4_ upon OER cycling, during which the surfaces become Ni‐ and O‐rich, which can promote the OER activity.

**Table 1:** Average Fe/Ni and O/M ratios in the bulk‐ and Ni‐rich regions of NiFe_2_O_4_ and Ni_1.5_Fe_1.5_O_4_ nanoparticles in the pristine state and after 10 and 500 CV cycles, and average Fe/Ni ratios, O/M ratios, and P concentrations in bulk‐ and P‐rich regions of NiFe_2_O_4_‐5P nanoparticles at the pristine state and after 10 and 500 cycles.

	Fe/Ni ratio ^a)^		O/M ratio ^b)^	
	Bulk	Ni‐rich	Bulk	Ni‐rich
NiFe_2_O_4_ nanoparticles				
Pristine	2.6 ± 0.2	–	1.09 ± 0.03	–
10 cycles	2.6 ± 0.2	1.5 ± 0.2	1.02 ± 0.04	1.00 ± 0.05
500 cycles	2.6 ± 0.2	1.5 ± 0.2	0.97 ± 0.03	1.04 ± 0.10

Ni_1.5_Fe_1.5_O_4_ nanoparticles				
Pristine	1.4 ± 0.1	1.1 ± 0.1	1.04 ± 0.04	1.07 ± 0.06
10 cycles	1.5 ± 0.1	0.8 ± 0.1	0.99 ± 0.05	0.99 ± 0.08
500 cycles	1.5 ± 0.1	0.7 ± 0.1	1.05 ± 0.05	0.96 ± 0.08

NiFe_2_O_4_‐5P nanoparticles						
	Fe/Ni ratio ^a)^		O/M ratio ^b)^		P / at.% ^c)^	
	Bulk	Ni‐P‐rich	Bulk	Ni‐P‐rich	Bulk	Ni‐P‐rich
Pristine	2.6 ± 0.1	2.2 ± 0.2	1.01 ± 0.05	1.16 ± 0.05	0.36 ± 0.02	1.37 ± 0.06
10 cycles	2.5 ± 0.2	2.2 ± 0.2	1.01 ± 0.03	1.06 ± 0.05	0.21 ± 0.02	1.11 ± 0.05
500 cycles	2.5 ± 0.2	2.0 ± 0.1	1.03 ± 0.04	1.00 ± 0.04	0.26 ± 0.01	1.02 ± 0.04

^a)^ The mathematical equation of $R\sqrt{{\left(\frac{\sigma_{a}}{a}\right)}^{2}+{\left(\frac{\sigma_{b}}{b}\right)}^{2}}$ is used to estimate the errors of Fe/Ni ratio, where R represents the average Fe/Ni ratio, a and b represent the average Fe and Ni concentration, and σ_a_ and σ_b_ represent the standard deviation of Co and Fe concentration of all reconstructed nanoparticles;

^b)^ The mathematical equation of $R\sqrt{{\left(\frac{\sigma_{c}}{c}\right)}^{2}+{\left(\frac{\sigma_{d}}{d}\right)}^{2}}$ is used to estimate the errors of Fe/Ni ratio, where R represents the average O to (Fe + Ni) ratio, c, and d represent the average O and (Fe + Ni) concentration, and σ_c_ and σ_d_ represent the standard deviation of O and (Fe+ Ni) concentration of all reconstructed nanoparticles;

^c)^ The mathematical equation of $\sqrt{\frac{c(100-c)}{N}}$ was used to calculate the error bar of P concentration, where c and N are the concentration and the total number of atoms within the fixed bin width profile of 0.4 nm, respectively.

For pristine NiFe_2_O_4_‐5P, the cross‐sectional atom map, depicted in Figure 4g, reveals that P (orange dots) is distributed mainly within the topmost 1–2 nm layer of the nanoparticles, yielding a high local concentration of ≈3–6 at.% (see additional APT data in Figure S6, Supporting Information). Interestingly, the P‐rich surfaces of the active NiFe_2_O_4_‐5P are also O‐enriched, with an O/M ratio of ≈1.2 (see Table 1 and compositional histogram Figure 5d), similar to the oxygen content of activated NiFe_2_O_4_ after OER cycling. These results give a strong indication that the active species on the surfaces of pristine NiFe_2_O_4_‐5P and activated NiFe_2_O_4_ (after, e.g., 100 cycles) have a higher concentration of oxygen than those in the bulk. In addition, the P‐ and O‐rich surfaces of pristine NiFe_2_O_4_‐5P are enriched with Ni and deficit in Fe with a slightly lower Fe/Ni ratio (≈2.2, see Table 1). Previous studies speculate that P atoms substitute for oxygen anions.^[^ ^6^, ^33^ ^]^ Our results show that P most likely occupies the Fe sites, resulting in the loss of Fe on the surface of pristine P‐doped NiFe_2_O_4_. This result agrees with the thermodynamic view that P substitution of the interstitial Fe sites is more energetically favorable than that of oxygen anions.^[^ ^34^ ^]^ Upon OER cycling, the averaged surface P concentration decreases to less than ≈2 at.% after 500 cycles (Figure 5e), in agreement with our XPS data (Figure 3l), suggesting a slow P leaching. Additionally, the O/M ratio of NiFe_2_O_4_‐5P decreases with increasing number of CV cycles, reaching a value of ≈1.0 after 500 cycles, similar to that in the bulk part (Table 1, Figures 4k,l and 5d). Given that the OER activity decreases slowly (Figure 2e,k), we speculate that the gradual loss of P and O from the surface of NiFe_2_O_4_‐5P possibly contributes to deactivation during OER cycling. Notably, the Fe/Ni ratio on the Ni‐P‐rich surfaces of NiFe_2_O_4_‐5P decreases, but only slightly, after 500 cycles; the Fe/Ni ratio is significantly higher than that of NiFe_2_O_4_ surfaces. This finding indicates that Fe does not dissolve much, or that dynamic dissolution and redeposition^[^ ^35^ ^]^ possibly occur in P‐doped NiFe_2_O_4_ under OER conditions.

In comparison, the pristine Ni_1.5_Fe_1.5_O_4_ nanoparticle surface is already enriched with Ni, with a Fe/Ni ratio of ≈1.1, lower than that in the bulk (≈1.4, see Figure 4p and Table 1 and more APT data in Figure S7, Supporting Information). In addition, three of the ≈20 pristine Ni_1.5_Fe_1.5_O_4_ nanoparticles investigated, were significantly enriched in Ni (≈30 at.%) and O (≈60 at.%) (Figure S7b, Supporting Information). The O/M ratio in these three Ni_1.5_Fe_1.5_O_4_ nanoparticles was ≈1.5, considerably higher than in the remaining pristine Ni_1.5_Fe_1.5_O_4_ nanoparticles. This confirms the presence of Ni‐rich (oxy)hydroxides, possibly explaining why two pairs of redox peaks were observed for Ni_1.5_Fe_1.5_O_4_ upon cycling (Figure 2l), as the Ni(II)↔Ni(III) transition in Ni_1.5_Fe_1.5_O_4_ and Ni‐based (oxy)hydroxide occurs at different potentials due to different atomic environments.^[^ ^8c,d^ ^]^ As OER proceeds, Fe dissolves from the Ni‐rich surfaces, accompanied by a slight O loss, thus yielding a Fe‐ and O‐depleted surface after 500 cycles (Table 1, Figures 4q,r and 5f,g). In summary, the surfaces of NiFe_2_O_4_, NiFe_2_O_4_‐5P, and Ni_1.5_Fe_1.5_O_4_ nanoparticles undergo clearly different compositional changes. Fe dissolves from the surfaces of all three nanoparticle systems but to a lesser extent on NiFe_2_O_4_‐5P, forming Ni‐rich surfaces on three Ni‐Fe spinels. Importantly, the oxygen content in the NiFe_2_O_4_ surface increases as OER proceeds. The oxygen concentration on the surfaces of activated NiFe_2_O_4_ and pristine NiFe_2_O_4_‐5P with enhanced OER activity is higher than those in the bulk regions of nanoparticles.

**Figure 6:**
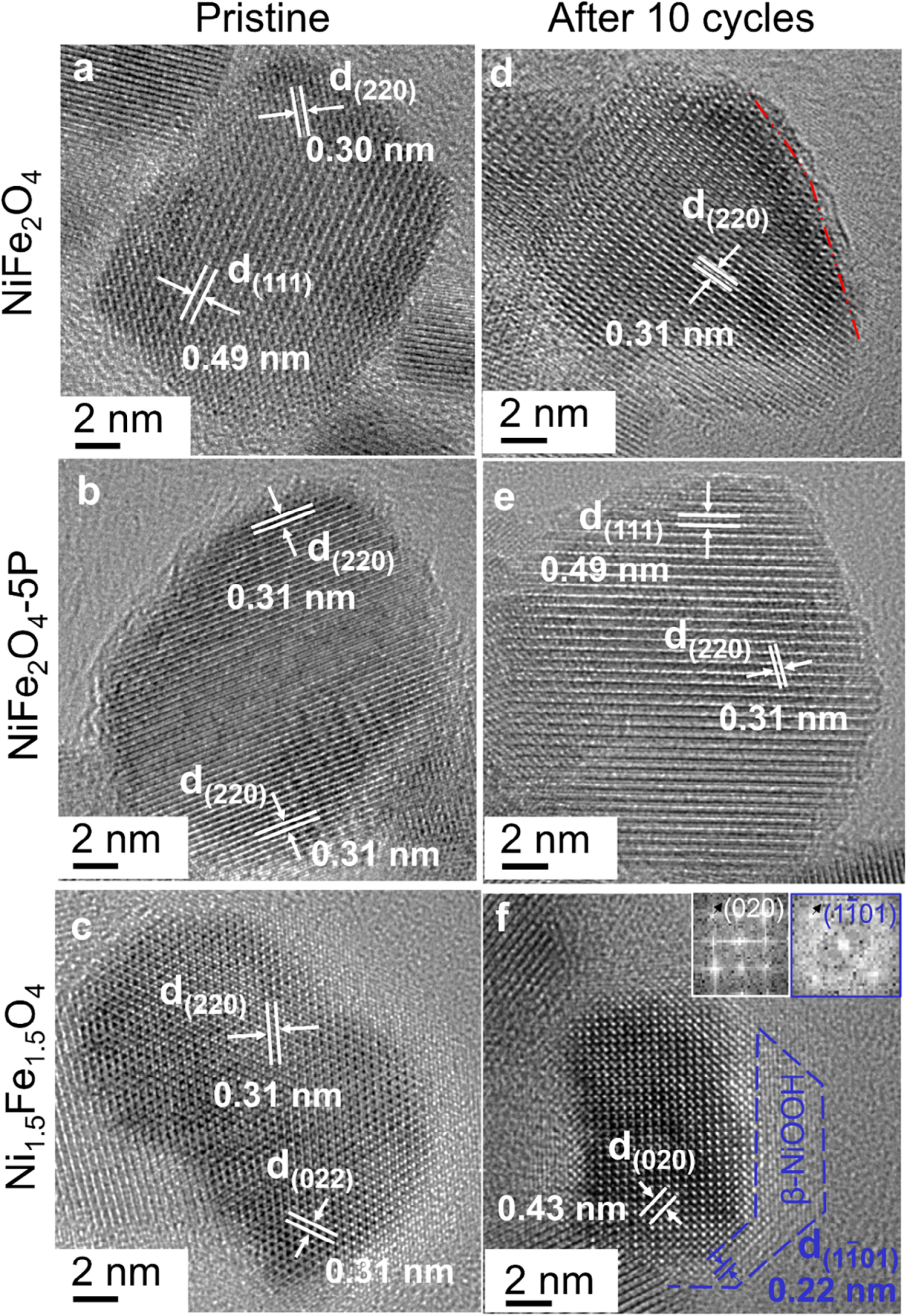
High‐resolution TEM images of NiFe_2_O_4_, NiFe_2_O_4_‐5P, and Ni_1.5_Fe_1.5_O_4_ nanoparticles a–c) in the pristine state and d–f) after 10 CV cycles under the OER conditions.

To examine whether the pronounced compositional changes are accompanied by surface structural changes, we employed high‐resolution TEM on the NiFe_2_O_4,_ NiFe_2_O_4_‐5P and Ni_1.5_Fe_1.5_O_4_ nanoparticles before and after 10 cycles of OER. **Figure** **6**a–c shows that the pristine NiFe_2_O_4,_ NiFe_2_O_4_‐5P and Ni_1.5_Fe_1.5_O_4_ have a cubic spinel structure (Fd $\bar{3}$ m) with a lattice constant of ≈8.46 Å. The top ≈1 nm surface of NiFe_2_O_4_‐5P is distinctly defective or amorphous, as its surface structure lacks lattice ridges (Figure 6b) compared to the crystalline surfaces of NiFe_2_O_4_ and Ni_1.5_Fe_1.5_O_4_ nanoparticles (Figure 6a,c). This explains why ECSA of pristine NiFe_2_O_4_‐5P is considerably larger than the other two (Figure S2d–f, Supporting Information). The observed ≈1 nm thick amorphous or defective surface of NiFe_2_O_4_‐5P persists after 10 CV cycles (Figure 6e). Interestingly, the surfaces of NiFe_2_O_4_ also become partially amorphous after 10 cycles (Figure 6d). In contrast, the surfaces of Ni_1.5_Fe_1.5_O_4_ remain crystalline, while new surface species are formed. The Fast Fourier filtered transform (FFT) image recorded from the blue dashed region (Figure 6f, right inset) shows that the motifs on the surface of Ni_1.5_Fe_1.5_O_4_ are aligned at 45° to the atomistic arrangement of the bulk spinel structure (Figure 6f, left inset). The reflection spots recorded from the surface region (Figure 6f, right inset) correspond to the β‐NiOOH phase (R $\bar{3}$ m, hexagonal). Based on this, we hypothesize that epitaxial growth of (Ni, Fe)OOH occurs on Ni_1.5_Fe_1.5_O_4_, with an orientation relationship of (010) Ni_1.5_Fe_1.5_O_4_//(1$\bar{1}$01) (Ni, Fe)OOH. This is in agreement with our XPS data, which shows that the surface of Ni_1.5_Fe_1.5_O_4_ after 100 CV cycles to be dominantly covered by hydroxide ions (Figure 3h), most likely due to the in situ surface transformation into (Ni, Fe)OOH during OER.

Our APT and TEM results show that the surfaces of activated NiFe_2_O_4_ and NiFe_2_O_4_‐5P are highly defective with a higher oxygen concentration (Table 1, Figure 4f,j), which is termed metal‐oxygen species in this study. Although the TEM and APT measurements were performed ex situ, our results most likely reveal the atom arrangements of the active species in the resting state. To interrogate the presence of the surface species and examine if they are formed from oxyhydroxides, we performed operando SERS measurements on NiFe_2_O_4_, NiFe_2_O_4_‐5P and Ni_1.5_Fe_1.5_O_4_ nanoparticles. The Raman spectra of pristine NiFe_2_O_4_, NiFe_2_O_4_‐5P, and Ni_1.5_Fe_1.5_O_4_ show similar peak features (Figure S8 and Table S2, Supporting Information), which are in good agreement with the literature of the spinels.^[^ ^36^ ^]^ We first interrogated the wavenumber region of 900–1150 cm^−1^ to examine the formation of active oxygen species, i.e., negatively charged superoxo species reported on NiFeO_x_ catalysts during OER.^[^ ^10b^ ^]^ The active oxygen species were hypothesized to be detected in pristine NiFe_2_O_4_‐5P and 100‐cycle NiFe_2_O_4_ samples. However, all samples exhibit peaks in the wavenumber region of 900–1150 cm^−1^ because these peaks also correspond to structural features of spinels (see Figure S9, Supporting Information). Thus, the presence of active oxygen species for pristine NiFe_2_O_4_‐5P and 100‐cycle NiFe_2_O_4_ spinels cannot unambiguously be confirmed by operando SERS. Instead, we focused on the ratio of characteristic peaks at 477 and 550 cm^−1^, which correspond to the Ni‐O bending and Ni‐O stretching, respectively. The peak ratio is often used to indicate the active or less active (oxy)hydroxides for OER.^[^ ^10^, ^22^, ^37^ ^]^

**Figure 7:**
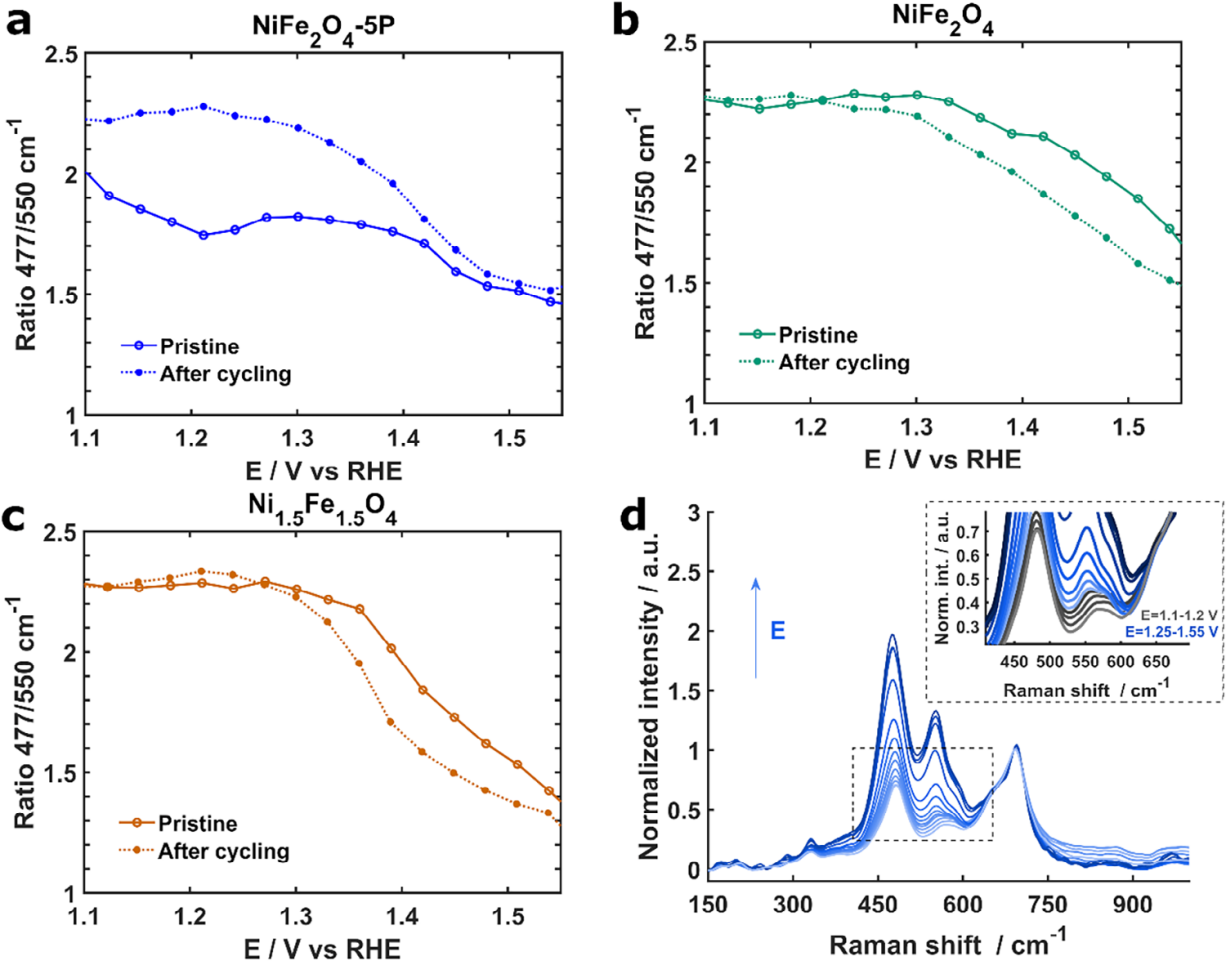
Changes in the ratio between the peaks at 477 and 550 cm^−1^ during operando surface enhanced Raman spectroscopy (SERS) measurements of a) pristine NiFe_2_O_4_‐5P and 50‐cycle NiFe_2_O_4_‐5P, b) NiFe_2_O_4_ and 100‐cycle NiFe_2_O_4_ c) Ni_1.5_Fe_1.5_O_4_ and 100‐cycle Ni_1.5_Fe_1.5_O_4_ at the potential range of 1.1–1.55 V versus RHE in 1 m KOH, d) original Raman spectra recorded during the operando SERS measurements of pristine NiFe_2_O_4_‐5P from 1.1 to 1.55 V versus RHE in the range of 150–1000 cm^−1^.

The dependence of the peak ratio of NiFe_2_O_4_‐5P, 50‐cycle NiFe_2_O_4_‐5P, NiFe_2_O_4_, 100‐cycle NiFe_2_O_4_, Ni_1.5_Fe_1.5_O_4,_ and 100‐cycle Ni_1.5_Fe_1.5_O_4_ on potential is summarized in **Figure** **7**a–c (see the original Raman spectra in Figure S10, Supporting Information). Interestingly, the peak at ≈550 cm^−1^ of all three pristine samples is broadened at 1.1–1.2 V versus RHE, which is likely associated with the cation redistribution in the spinel structure. Specifically, more cations are redistributed to the octahedral sites and fewer cations to the tetrahedral sites,^[^ ^38^ ^]^ as exemplified by the spectra of pristine NiFe_2_O_4_‐5P (Figure 7d). As the potential increases to 1.25 V versus RHE, distinct peaks corresponding to NiOOH appear (blue spectra), suggesting the in situ transformation from spinels to oxyhydroxides. The ratio of peaks at 477 and 550 cm^−1^ decreases at above 1.25 V versus RHE due to the initial higher ratio value induced by the spinel structure. Notably, the ratio of pristine NiFe_2_O_4_‐5P at 1.3 V versus RHE is ≈1.8, significantly lower than that of pristine NiFe_2_O_4_ and Ni_1.5_Fe_1.5_O_4_ (≈2.3) (Figure 7a–c). In comparison, the peak ratio of 50‐cycle NiFe_2_O_4_‐5P at 1.3 V versus RHE increases to 2.3, suggesting that the surfaces of NiFe_2_O_4_‐5P undergo substantial structural transformation. Additionally, the curves of 100‐cycle NiFe_2_O_4_ and 100‐cycle Ni_1.5_Fe_1.5_O_4_ at above 1.4 V versus RHE shift to lower potentials compared to those in the pristine state, suggesting that, after 100 cycles, active species are formed at lower the potentials (Figure 7b–c). As the potential increases to ≈1.53 V versus RHE, the peak ratio of all samples decreases to ≈1.5 (Figure 7a–c). The peak ratio of 100‐cycle Ni_1.5_Fe_1.5_O_4_ at ≈1.53 V versus RHE is lower (1.3–1.4) than that of 100‐cycle NiFe_2_O_4_ and 50‐cycle NiFe_2_O_4_‐5P (≈1.5). The typical peak ratio of α‐Ni(OH)_2_/γ‐NiOOH is ≈1.55 and it decreases to ≈1.4 after transformation to β‐Ni(OH)_2_/β‐NiOOH and ≈1.3 for β‐(Ni, Fe)(OH)_2_/β‐(Ni, Fe)OOH.^[^ ^10^, ^22^, ^37^ ^]^ Based on this, we speculated that the surface of 100‐cycle Ni_1.5_Fe_1.5_O_4_ might transform into β‐(Ni, Fe)OOH, while γ‐(Ni, Fe)OOH might be formed on the surfaces of 100‐cycle NiFe_2_O_4_ and 50‐cycle NiFe_2_O_4_‐5P at 1.53 V versus RHE. However, the peaks from the T_2_ _g_ modes of the Ni spinels in our study are also at 460–471 and 530–550 cm^−1^, which overlap with those of Ni(OH)_2_/NiOOH. Such overlap prevents us from directly comparing our peak ratios with those reported in the literature.^[^ ^10^, ^22^, ^37^ ^]^

Overall, our multimodal approach unprecedentedly provides atomic‐scale details into the different surface species formed on NiFe_2_O_4,_ NiFe_2_O_4_‐5P, and Ni_1.5_Fe_1.5_O_4_ after OER, whereby their formation mechanisms can be elucidated on Ni‐Fe spinel surfaces during OER. Despite ex situ XPS, TEM, and APT measurements, our results indicate the compositional and structural characteristics of the active species in the resting state formed on NiFe spinels. Specifically, one new observation from our APT and TEM data is that the surfaces of activated NiFe_2_O_4,_ and pristine NiFe_2_O_4_‐5P are amorphous with a higher oxygen concentration than the bulk part of nanoparticles (Table 1, Figures 4f and 5j). In addition, the XPS data of activated NiFe_2_O_4,_ and pristine NiFe_2_O_4_‐5P reveals the presence of additional Ni 2p spectrum at ≈853 eV and a shift of O 1s spectrum to ≈528.4 eV, which was ascribed to the adsorption of oxygen‐containing species at the defects in NiO.^[^ ^25^ ^]^ These results infer that the active metal‐oxygen species are highly disordered or defective, possibly like superoxo species^[^ ^10b^ ^]^ in resting state (due to the higher oxygen concentration on surfaces). However, we cannot make any conclusive statement since our operando SERS measurement did not confirm the presence of negatively charged superoxo species on the surfaces of activated NiFe_2_O_4,_ and NiFe_2_O_4_‐5P due to the peak overlap with structural features of Ni‐Fe spinels. Nevertheless, such surface species with higher oxygen concentration are formed on activated NiFe_2_O_4,_ and NiFe_2_O_4_‐5P upon OER cycling, but not on Ni_1.5_Fe_1.5_O_4_. One major difference between NiFe_2_O_4,_ NiFe_2_O_4_‐5P, and Ni_1.5_Fe_1.5_O_4_ is that the Fe/Ni ratio on the surfaces of pristine NiFe_2_O_4_ and NiFe_2_O_4_‐5P is ≈2.6, while it is only ≈1.1 on the surface of Ni_1.5_Fe_1.5_O_4_ (see our APT data in Table 1, Figures 4, 5). These results suggest that more Fe surrounding Ni (with a Fe/Ni ratio of ≈2.6) in Ni‐based spinels is essential for forming active species. This contrasts with the Fe/Ni ratio in Ni‐based oxyhydroxides or hydroxides, where adding small amounts of Fe (with a Fe/Ni ratio of ≈0.25) enhances their OER activity the most.^[^ ^8d^ ^]^ Our findings suggest that how Fe affects the OER activity of Ni‐based spinels differs from that of the Ni‐based (oxy)hydroxides that need significantly less Fe to improve the OER activity.

Essentially, the Ni‐Fe spinels with higher Fe/Ni ratios or P doping yield a lower averaged Ni oxidation state. Previous work^[^ ^10^, ^39^ ^]^ proposed that the formation of high oxidation state Ni(IV), assisted by Fe(III) acting as the Lewis acid (electron acceptor), is essential for OER activity since Ni(IV) oxo radicals promote O─O bond formation. Our XPS observation of the low oxidation state Ni was obtained from ex situ measurements. A previous study^[^ ^40^ ^]^ also observed that the active species contains Ni at a lower oxidation state (+2), but the authors speculated that Ni(IV) rapidly converted to Ni(II). This Ni(IV) to Ni(II) conversion would prevent the accumulation of Ni(III)/Ni(IV) species to a detectable level; this might also be the case in our study. Thus, the presence of Ni(IV) during OER of NiFe_2_O_4_ and NiFe_2_O_4_‐5P cannot be excluded entirely. Despite this, our XPS data (Figure 3a,b) suggest that some of the Ni sites have lower Ni oxidation (<+2), implying that the active metal‐oxygen species contain Ni sites with high discharge ability and reducibility. Thus, we speculate that the amounts of Ni sites at low oxidation state (Ni, II) and the reducibility of Ni(II)↔Ni(III) are critical for the active surface species. The amounts of such low oxidation state Ni sites are associated with the Fe/Ni ratio in the Ni‐Fe mixed spinels. The higher Fe/Ni ratios in Ni‐Fe spinels are needed to induce a low oxidation state Ni. Additionally, Fe may act as an electron donor, promoting and stabilizing the active species. Given that Fe 2p spectra exhibit a significant overlap with the Ni LMM Auger peak, the oxidation state change of Fe during OER cycling cannot resolved by our XPS data. However, to maintain Ni sites' high discharge ability and reducibility, elements like Fe or P with a higher affinity for losing electrons are thought to be needed for active metal‐oxygen species formed on the Ni‐based spinels. This is consistent with DFT simulations proposed that Fe in NiFe_2_O_4_^[^ ^11^ ^]^ is more active than Ni toward OER, or both Fe(IV) and Ni(IV) contribute to optimal OER performance^[^ ^9^, ^10^ ^]^ as the high spin Fe(IV) in (Ni, Fe)OOH aids the efficient generation of an active O radical intermediate and Ni(IV) catalyzes the subsequent O─O bond coupling. Thus, Fe, Ni, and P jointly contribute to the improved OER activity of activated NiFe_2_O_4_ and P‐doped NiFe_2_O_4_. The formation of active species in Ni‐based spinels requires a high Fe/Ni ratio (≈2) or elements like P with a higher affinity of losing electrons to maintain the reducibility of Ni.

In addition to a high Fe/Ni ratio, forming active metal‐oxygen species might require a disordered arrangement on the Ni‐Fe spinel surface. After P doping, the NiFe_2_O_4_‐5P surfaces become amorphous (Figure 6b), whereby higher ECSAs are generated to promote the activity and charge transfer kinetics (Figure S2d–l, Supporting Information). The defective surfaces of P‐doped NiFe_2_O_4_ are thought to promote the adsorption of hydroxyl ions, giving rise to a significant improvement of OER activity compared to pristine NiFe_2_O_4_. For NiFe_2_O_4_, the surface becomes highly defective after OER cycling (Figure 6d), as seen in decreases in the Tafel slope, reaching values similar to pristine NiFe_2_O_4_‐5P (Figure 2g). We speculate that continuous Fe and/or P dissolution, and potentially dissolution/redeposition^[^ ^35^ ^]^; for example, possibly promote the surface reconstruction and transformation by creating defects such as cation vacancies. These defective sites enhance the adsorption or deprotonation of hydroxyl ions, likely leading to the formation of active metal‐oxygen species during OER cycling, which enables fast redox dynamics according to the transformation liability mode proposed by Tschulik and her coworkers.^[^ ^41^ ^]^ Additionally, P dissolution, likely in the form of phosphate anions, was found to reduce Gibbs free energy of the peroxo species formation of NiFe layered double hydroxide^[^ ^42^ ^]^ or lower the adsorption of peroxo species on faceted CoFe_2_O_4_ spinel.^[^ ^43^ ^]^ Therefore, P dissolution not only creates more defective sites on P‐doped NiFe_2_O_4_ nanoparticle surfaces, but its presence at the electrode/electrolyte interface possibly also promotes the intermediate species formation. Notably, although Fe dissolution also occurred for Ni_1.5_Fe_1.5_O_4_, no active metal‐oxygen species were observed. This suggests that Fe dissolution is likely not the dominant factor, but a higher Fe/Ni ratio (>2) in the pristine Ni‐based spinels is favorable to form active metal‐oxygen species. For Ni_1.5_Fe_1.5_O_4_, oxyhydroxides dominate the catalyst surface after OER, as revealed by our TEM data (Figure 6f). While oxyhydroxides are also formed on the surfaces of NiFe_2_O_4_ and NiFe_2_O_4_‐5P upon OER cycling, under operation conditions, they might differ from that formed on the Ni_1.5_Fe_1.5_O_4_ since our operando SERS results reveal a lower peak ratio at 460–471 and 530–550 cm^−1^ for 100‐cycle Ni_1.5_Fe_1.5_O_4_ (Figure 7a–c). Given that the structural features of Ni oxyhydroxides and Ni‐based spinel overlap at ≈555 cm^−1^, it is difficult to confirm the types of Ni oxyhydroxides formed on the three samples by relating our findings with those of previous work.^[^ ^10^, ^22^, ^37^ ^]^ Nevertheless, the Tafel slopes of 100‐cycle NiFe_2_O_4_ and 10‐cycle NiFe_2_O_4_‐5P are ≈40 mV dec^−1^, significantly lower than that of 100‐cycle Ni_1.5_Fe_1.5_O_4_ (≈90 mV dec^−1^). This suggests that the active metal‐oxygen species formed on 100‐cycle NiFe_2_O_4_ and 10‐cycle NiFe_2_O_4_‐5P induce faster charge transfer kinetics compared to the oxyhydroxides grown on Ni_1.5_Fe_1.5_O_4_, possibly via different OER reaction mechanisms, as previously proposed,^[^ ^44^ ^]^ or by different hydroxide coverage. In forthcoming research, isotope‐labeled NiFe spinels will be prepared to investigate OER reaction mechanisms by, e.g., operando differential electrochemical mass spectroscopy.

## 3 Conclusion

In summary, our multimodal method provides atomic‐scale insights into the surface species formed on NiFe_2_O_4_, P‐doped NiFe_2_O_4,_ and Ni_1.5_Fe_1.5_O_4_ during OER. ≈1 nm thick amorphous metal‐oxygen species with higher oxygen concentration are formed on the surfaces of activated NiFe_2_O_4_ and P‐doped NiFe_2_O_4_ nanoparticles. P doping in NiFe_2_O_4_ is thought to induce the defective surface arrangement for forming active metal‐oxygen species at the onset of OER, contributing to the high OER activity of P‐doped NiFe_2_O_4_. Additionally, the formation of such species in Ni‐based spinels is thought to require a high Fe/Ni ratio (≈2), since the stabilization of active species needs elements like Fe and P with higher affinity to lose electrons to maintain the reducibility of Ni(II)↔Ni(III). The active species grown on activated NiFe_2_O_4_ and P‐doped NiFe_2_O_4_ yield a significantly lower Tafel slope (≈40 mV dec^−1^) compared to oxyhydroxides grown on Ni_1.5_Fe_1.5_O_4_ (≈90 mV dec^−1^), promoting the OER reaction kinetics. Overall, this study provides unprecedented atomic‐scale insights into the chemical nature of surface species in Ni‐spinels and reveals the effects of P, Fe, and Ni on the formation of surface species toward OER.

## 4 Experimental Section

### Chemicals

Iron nitrate (Fe(NO_3_)_3_·9H_2_O, ACS reagent), nickel nitrate (Ni(NO_3_)_2_·6H_2_O, ACS reagent), ammonia (NH_3_·H_2_O), polyethene glycol (PEG, Mn = 400) and sodium hypophosphite (NaH_2_PO_2_·H_2_O, ACS reagent) were purchased from Sinopharm Chemical Reagent. KOH (ACS reagent), KOD (40wt.% in D_2_O, 98 at.% D), D_2_O (99.9 at.% D), and 5 wt.% Nafion solutions were purchased from Sigma–Aldrich. Isopropanol (ACS reagent) was purchased from VWR. All reagents were used without further purification.

### Material Synthesis

Three Ni‐Fe oxides were prepared by dropwise the complexing agent of ammonia into the precursor, following a standard hydrothermal process. As for NiFe_2_O_4_ nanoparticles preparation, the precursor solution was obtained by mixing 3.2 g of Fe(NO_3_)_3_·9H_2_O, 1.2 g of Ni(NO_3_)_2_·6H_2_O, 0.2 g of polyethylene glycol (PEG, Mn = 400), and 40 mL of ultrapure water (0.055 µS cm^−1^) under magnetic stirring for 30 min. 10 mL of the diluted ammonia (1:1 v/v ammonia/water) was subsequently added into this precursor solution under vigorous stirring for 30 min. Then the as‐prepared brown precipitate was transferred into a 100 mL Teflon autoclave, maintained at 180 °C for 3 h. The final dark brown powder was obtained after washing with ultrapure water and drying at 80 °C. Preparations of the Ni_2_FeO_4_ and Ni_1.5_Fe_1.5_O_4_ samples used identical procedures as the NiFe_2_O_4_ except for the difference in the weight ratio of Fe(NO_3_)_3_·6H_2_O and Ni(NO_3_)_2_·6H_2_O in the mixed precursor solution. Phosphorus‐doped NiFe_2_O_4_ nanoparticles were synthesized by calcinating the mixture of as‐prepared NiFe_2_O_4_ and NaH_2_PO_2_·H_2_O. For the preparation of NiFe_2_O_4_‐5P, 40 mg of NaH_2_PO_2_·H_2_O (8 and 80 mg for NiFe_2_O_4_‐1P and NiFe_2_O_4_‐10P, respectively) and 250 mg of as‐prepared NiFe_2_O_4_ nanoparticles were mixed and ground together and then heated in the tube furnace at 300 °C for 2 h under nitrogen flow of 30 mL min^−1^. After cooling to room temperature, the final product was collected by the magnet, washed several times with ultrapure water, and dried in an oven at 80 °C for 12 h.

### Material Characterization

The XRD data of three Ni‐Fe oxides were collected on Bruker Discover D8 with a θ‐θ geometry and a Lynxeye‐1D detector and Cu Kα radiation (*λ* = 0.15406 nm, 40 kV, 40 mA). The crystal structure of NiFe_2_O_4_ and P‐doped NiFe_2_O_4_ nanoparticles were studied on Rigaku Ultima IV diffractometer with Cu Kα radiation (*λ* = 0.15418 nm). All XRD data were recorded in the 2*θ* range of 10 ≈ 90° with a scanning speed of 1.2 °/min and a step size of 0.02°. The morphologies and size distributions of these electrocatalysts were investigated on an aberration‐corrected JEOL JEM‐2200FS, operated at 200 kV. TEM samples were obtained by dropping the suspensions of the nanoparticles in anhydrous ethanol on carbon‐supported Cu grids and drying at room temperature. XPS experiments were performed on a VersaProbe II (Ulvac‐Phi) with a monochromatic Al X‐ray source (1486.6 eV), operated at 15 kV and 13.2 W with a 45° emission angle between the analyzer and the substrate surface. Software of CasaXPS was used to perform XPS data analysis and the carbon located at 284.8 eV was used to calibrate the binding energies of samples. For APT measurements, APT specimens of NiFe_2_O_4_, Ni_1.5_Fe_1.5_O_4_, and NiFe_2_O_4_‐5P nanoparticles were prepared by coupling nanoparticle encapsulation and standard FIB lift‐out method. In this study, Co electrodeposition was applied to encapsulate pristine NiFe_2_O_4_ and Ni_1.5_Fe_1.5_O_4_ nanoparticles, which is performed at a constant voltage of – 0.8 V (versus Ag/AgCl) for 120 s in the Co ion solution including 2.4 g of CoCl_2_·6H_2_O, 0.6 g of H_3_BO_3_, and 20 mL of D_2_O. To investigate the compositional evolution of catalysts after the OER processes, the corresponding APT specimens were prepared using the same procedures, expecting the CV treatments before Co deposition. The CV experiments were performed on glass carbon plates covered with nanoparticle catalysts. APT data were collected on CAMECA LEAP 5000XR instrument using laser pulsing mode at a specimen temperature of 57 K, laser energy of 30 pJ, pulse frequency of 125 kHz, and detection rates of 0.5, and commercial IVAS 3.8.10 software was applied to APT data analysis.

Operando Raman spectroscopy measurements were collected using a Raman Microscope (Alpha‐300R confocal Raman microscope – WITec, GmbH) equipped with a laser source of 532 nm. The laser power was set 1 mW for all the experiments and spectra were registered with an integration time of 1s. For a better comparison of the results the intensity of all Raman spectra has been normalized to the A_1_ _g_ peak (≈690 cm^−1^). To conduct ex situ Raman experiments, the catalyst ink was deposited on a glassy carbon plate and dried under N_2_, and an air 50x objective (Zeiss LD EC Epiplan‐Neofluar 50x / 0.55 HD) was employed to register the spectra. For operando measurements, a 3D printed home‐made cell of polyvinylidene fluoride (PVDF) was used with a 63x immersion objective (Zeiss W Plan‐Apochromat 63x/1.0 VIS‐IR). The catalyst ink was deposited on a gold electrode, which serves as a working electrode, and carbon rod and Ag/AgCl (3M KCl) were used as counter and reference electrodes, respectively. The electrochemical experiments were performed with a potentiostat PalmSens 4, coupled to the Raman Microscope. LSV experiments were performed with a scan rate of 10 mV s^−1^ in 1.0 m KOH from 0 to 0.8 V (versus Ag/AgCl), while Raman spectra were collected simultaneously each second. In the CV treatment of cycled samples, the potential was scanned from 0 to 0.65 V (versus Ag/AgCl) at a scan rate of 50 mV s^−1^. All potentials were corrected to RHE.

### Electrochemical Measurements

Electrocatalytic performances of the mixed Ni‐Fe oxides were evaluated on a rotating disk electrode system (RDE, ALS RRDE‐3A) with a Biologic potentiostat (SP300). The catalyst ink was prepared by dispersing 5 mg of nanoparticle powder into 1 mL mixed solvent containing 705 µl of ultrapure water, 250 µL of isopropanol, and 45 µL of 5 wt.% Nafion solution under ultrasonication for 30 min. Subsequently, the final working electrode was obtained after drying 10 µL of this catalyst dispersion on the cleaned glassy carbon disk (5 mm diameter, 0.19625 cm^2^), and Pt wire and Ag/AgCl (3 m KCl) served as the counter and reference electrodes, respectively. The LSV curves were recorded with a scan rate of 10 mV s^−1^ in oxygen‐saturated 1.0 m KOH from 0 to 0.8 V (versus Ag/AgCl) at a rotating speed of 1600 rpm. The CV measurements were performed at a scan rate of 50 mV s^−1^ from 0 to 0.65 V (versus Ag/AgCl). The electrochemical surface areas (ECSAs) of the catalysts were estimated by CV (details are given in Figure S2, Supporting Information). CVs were performed in the potential range from −0.05 to 0.05 V (versus Ag/AgCl) at varying rates from 25 to 125 mV s^−1^ in 1.0 m KOH. To investigate the compositional changes after CV treatment by APT characterization, the corresponding CV tests were performed in 1.0 m KOD (in D_2_O) solution at a scan rate of 50 mV s^−1^ from 0 to 0.65 V (versus Ag/AgCl).

## Conflict of Interest

The authors declare no conflict of interest.

## Supporting information



Supporting Information

## Data Availability

The data that support the findings of this study are available from the corresponding author upon reasonable request.
