# Supplementary material for: Unveiling Surface Species Formed on Ni‐Fe Spinel Oxides During the Oxygen Evolution Reaction at the Atomic Scale
Source: Adv Sci (Weinh). 2025 Mar 31;12(25):2501967. doi: 10.1002/advs.202501967 (PMC12224984; doi:10.1002/advs.202501967)
Supplement: Supplementary file 1 — Supporting Information [file ADVS-12-2501967-s001.pdf]

ADVANCED SCIENCE
Open Access

Supporting Information

for *Adv. Sci.*, DOI 10.1002/advs.202501967

Unveiling Surface Species Formed on Ni-Fe Spinel Oxides During the Oxygen Evolution Reaction at the Atomic Scale

Weikai Xiang, Sheila Hernandez, Pouya Hosseini, Fan Bai, Ulrich Hagemann, Markus Heidelmann and Tong Li**

Supporting Information of

Unveiling surface species formed on Ni-Fe spinel oxide nanoparticles during the oxygen evolution reaction at the atomic scale

Weikai Xiang[a], Sheila Hernandez[b]*, Pouya Hosseini[c], Fan Bai[a], Ulrich Hagemann[d], Markus Heidelmann[d], Tong Li[a]*

a Faculty of Mechanical Engineering, Atomic-scale Characterisation, Ruhr-Universität Bochum, Universitätsstraße 150, 44801 Bochum, Germany

b Faculty of Chemistry and Biochemistry, Analytical Chemistry II, Ruhr-Universität Bochum, Universitätsstraße 150, 44801 Bochum, Germany

c Max-Planck-Institut für Nachhaltige Materialien GmbH, Max-Planck-Straße 1, Düsseldorf 40237, Germany

d Interdisciplinary Center for Analytics on the Nanoscale (ICAN) and Center for Nanointegration Duisburg-Essen (CENIDE), University of Duisburg-Essen, Carl-Benz-Straße 199, 47057 Duisburg, Germany

Corresponding Author: tong.li@rub.de and sheila.hernandez@rub.de

Keywords: water splitting, Raman spectroscopy, atom probe tomography, active species, oxyhydroxides, OER

Table S1. The chemical formula of P-doped $NiFe_2O_4$ nanoparticles calculated from ICP-MS results.

Sample	Mass /mg	Volume /ml	Ni /ppm	Fe /ppm	P /ppm	Ni /at. %	Fe /at. %	P /at. %	Chemical Formula
$NiFe_2O_4$	4.4	50	19.93	44.43	0	13.0	30.5	0.0	$Ni_{0.9}Fe_{2.1}O_4$*
$NiFe_2O_4$-1P	5.2	50	22.3	50.16	0.718	11.8	27.8	0.7	$Ni_{0.9}Fe_{2.1}P_{0.1}O_4$*
$NiFe_2O_4$-5P	4.8	50	20.23	46.05	2.745	11.7	28.0	3.0	$Ni_{0.8}Fe_{2.0}P_{0.2}O_4$*
$NiFe_2O_4$-10P	4.2	50	13	29.28	2.881	11.4	27.1	4.3	$Ni_{0.8}Fe_{1.9}P_{0.3}O_4$*

*As the exact oxygen content could not be obtained by ICP-MS, all chemical formulas are calculated from the spinel structure of M_3O_4.

Table S2. Raman peaks position and assignment for the Raman spectra of the different catalysts deposited on glassy carbon.

Raman shift / cm^{-1}			Assignment [1]
$Ni_{1.5}Fe_{1.5}O_4$	$NiFe_2O_4$	$NiFe_2O_4$-5P	
193	196	196	T_{2g} (1)
302	310	307	E_g
462	465	471	T_{2g} (2)
532	540	549	T_{2g} (3)
672	680	680	A_{1g}

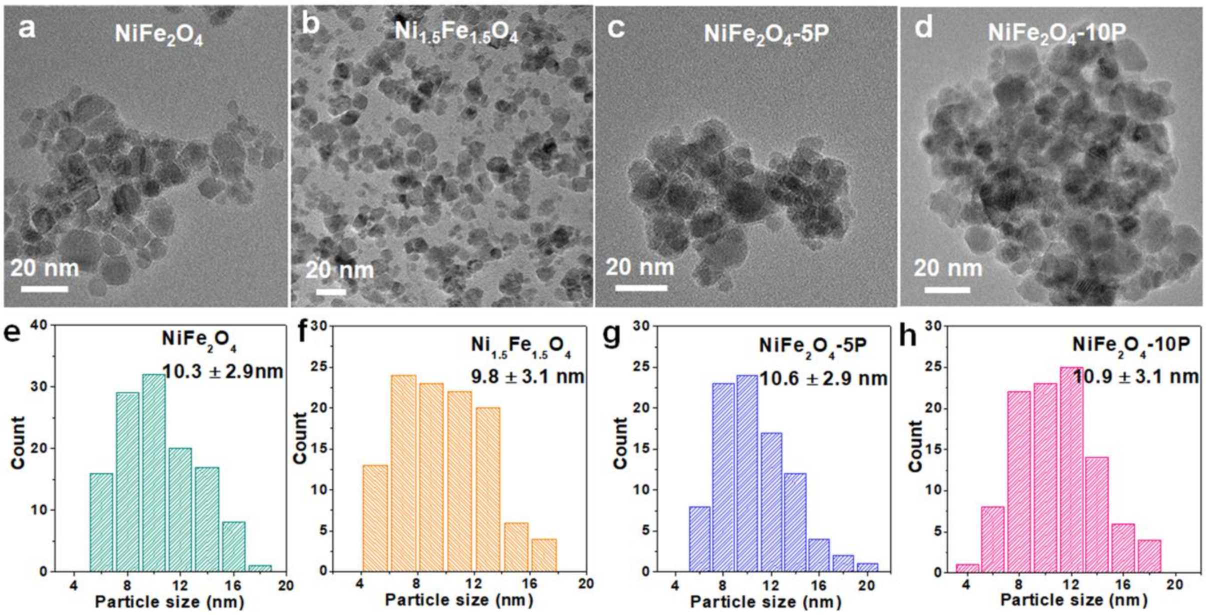

Figure S1. Characterizations of morphology and particle size. (a-d) TEM images, (e-h) corresponding size distributions of pristine $NiFe_2O_4$, $Ni_{1.5}Fe_{1.5}O_4$, $NiFe_2O_4$-5P and $NiFe_2O_4$-10P nanoparticles. The error bar in (e-h) is the standard deviation (SD) calculated using Origin software, and the corresponding mathematical equation is $SD = \sqrt{\frac{\sum_{i=1}^{n}(x_i - \bar{x})^2}{n-1}}$.

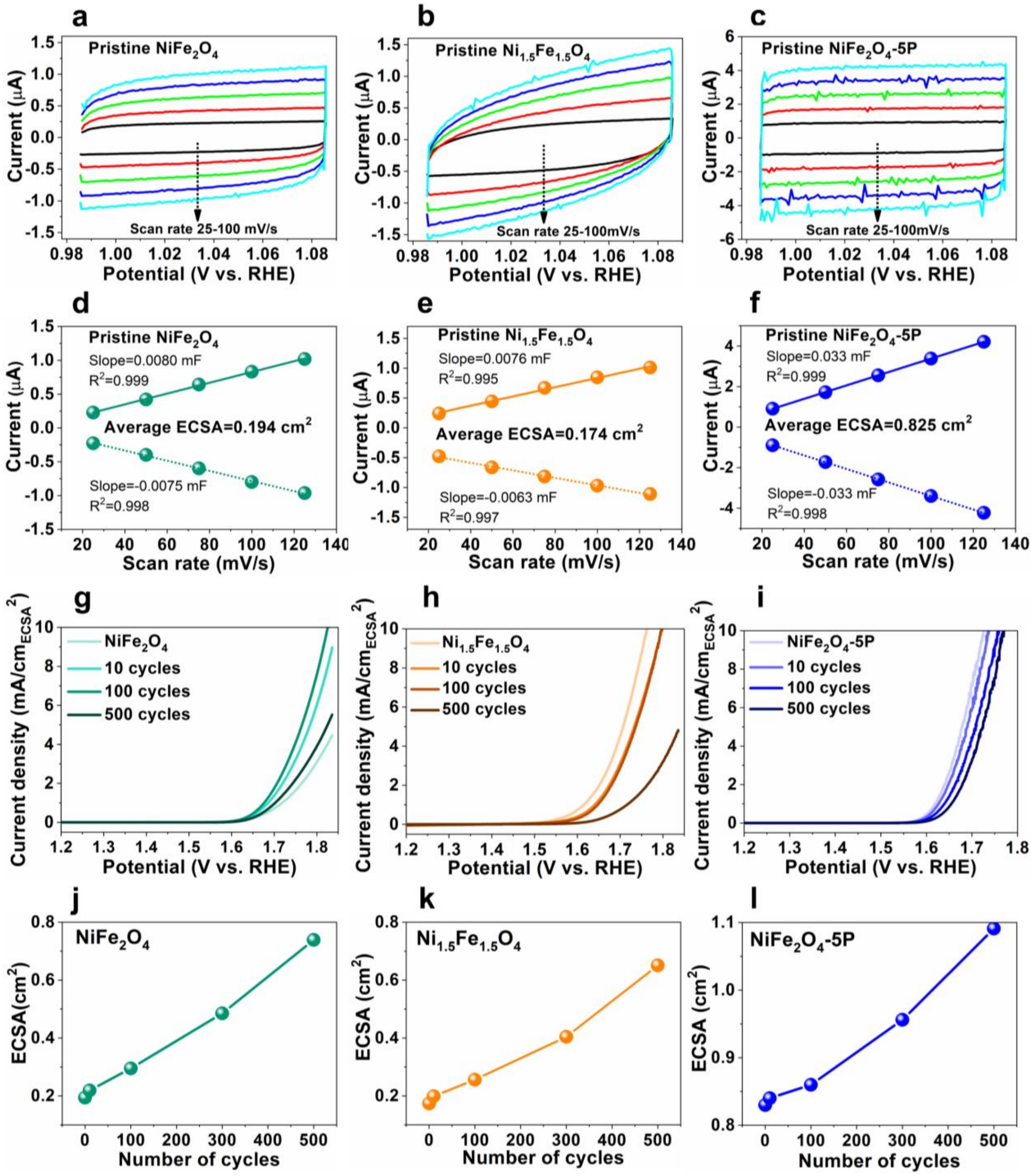

Figure S2. The electrochemical surface areas (ECSAs) and electrochemical activity of catalysts. (a-c) CV curves recorded in the non-Faradic potential window of - 0.05 ~ 0.05 V (vs. Ag/AgCl) at varying rates from 25 to 125 mV/s, (d-f) corresponding double-layer capacitances (Cdl) curves that are derived from the CVs of pristine $NiFe_2O_4$, $Ni_{1.5}Fe_{1.5}O_4$, and $NiFe_2O_4$-5P nanoparticles in (a-c), (g-i) LSV curves normalized by ECSA of $NiFe_2O_4$, $Ni_{1.5}Fe_{1.5}O_4$, and $NiFe_2O_4$-5P nanoparticles at the pristine state and after 10, 100, and 500 cycles. The average of the absolute value of the slopes of solid and dashed lines in (d-f) is regarded as the C_{dl}. The ECSA of a catalyst sample is calculated as the equation of ECSA=C_{dl}/C_s, where the general specific capacitance of C_s is 0.040 mF/cm^2 in 1 M KOH. These results indicate that $NiFe_2O_4$ and $Ni_{1.5}Fe_{1.5}O_4$ nanoparticles have a similar ECSA, but a remarkable increase in the ECSA is observed after P doping. Besides, P-doping also enhance the OER stability as shown in (g-i). (j-l) ECSAs measurements after 10, 100 and 500 CV cycles by using the same method described in (d-f).

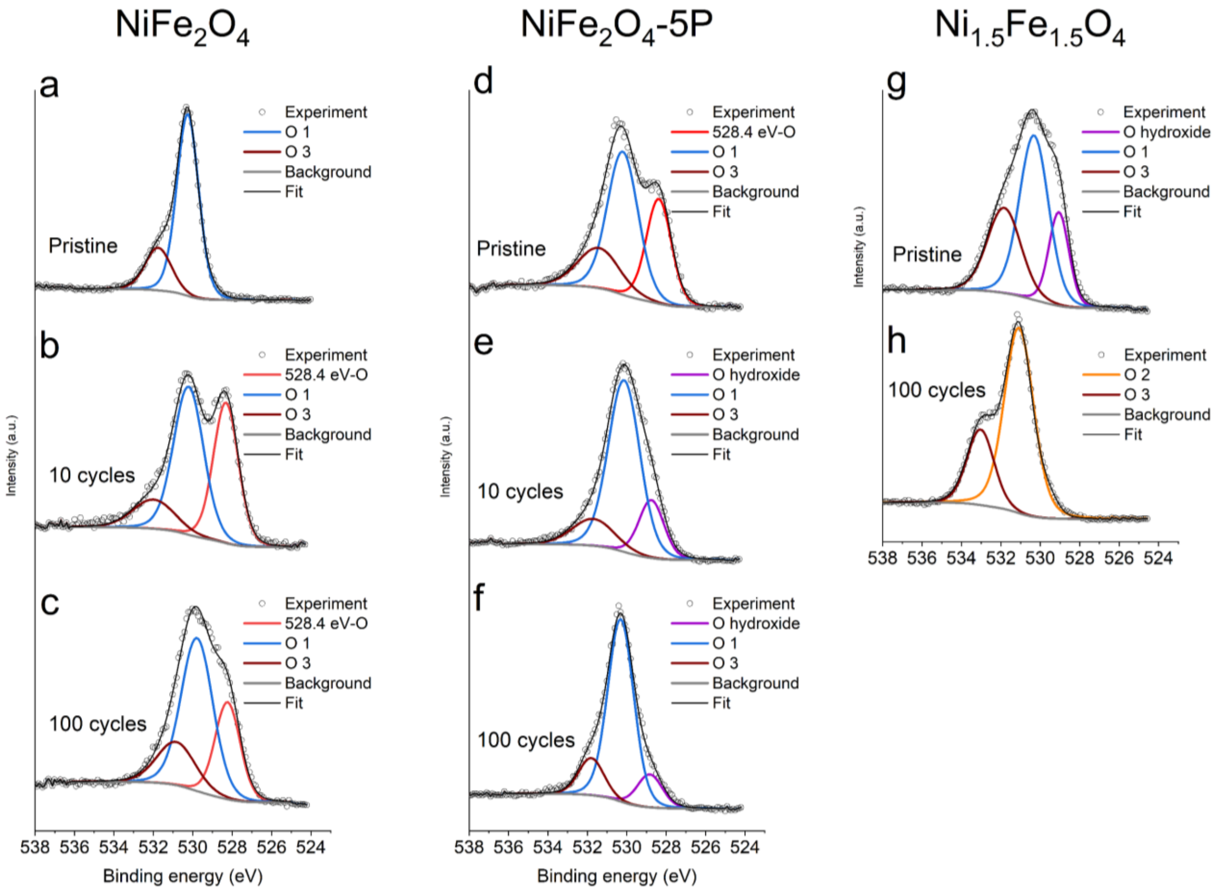

Figure S3. X-ray photoemission spectroscopy (XPS) spectra of O 1s of (a-c) $NiFe_2O_4$, (d-f) $NiFe_2O_4$-5P in the pristine state and after 10 and 100 CV cycles, (g-h) $Ni_{1.5}Fe_{1.5}O_4$ nanoparticles in the pristine state and after 100 CV cycles. The O1 peak, positioned at approximately 529.5 - 530.0 eV, corresponds to the lattice oxygen in the metal oxide (Ni– and Fe–O). The weak O3 peak, positioned at approximately 532 eV, is often attributed to unavoidable physically adsorbed or residual water molecules and/or carbon–oxygen bonds from contaminated organic carbon during sample transfer.[2]

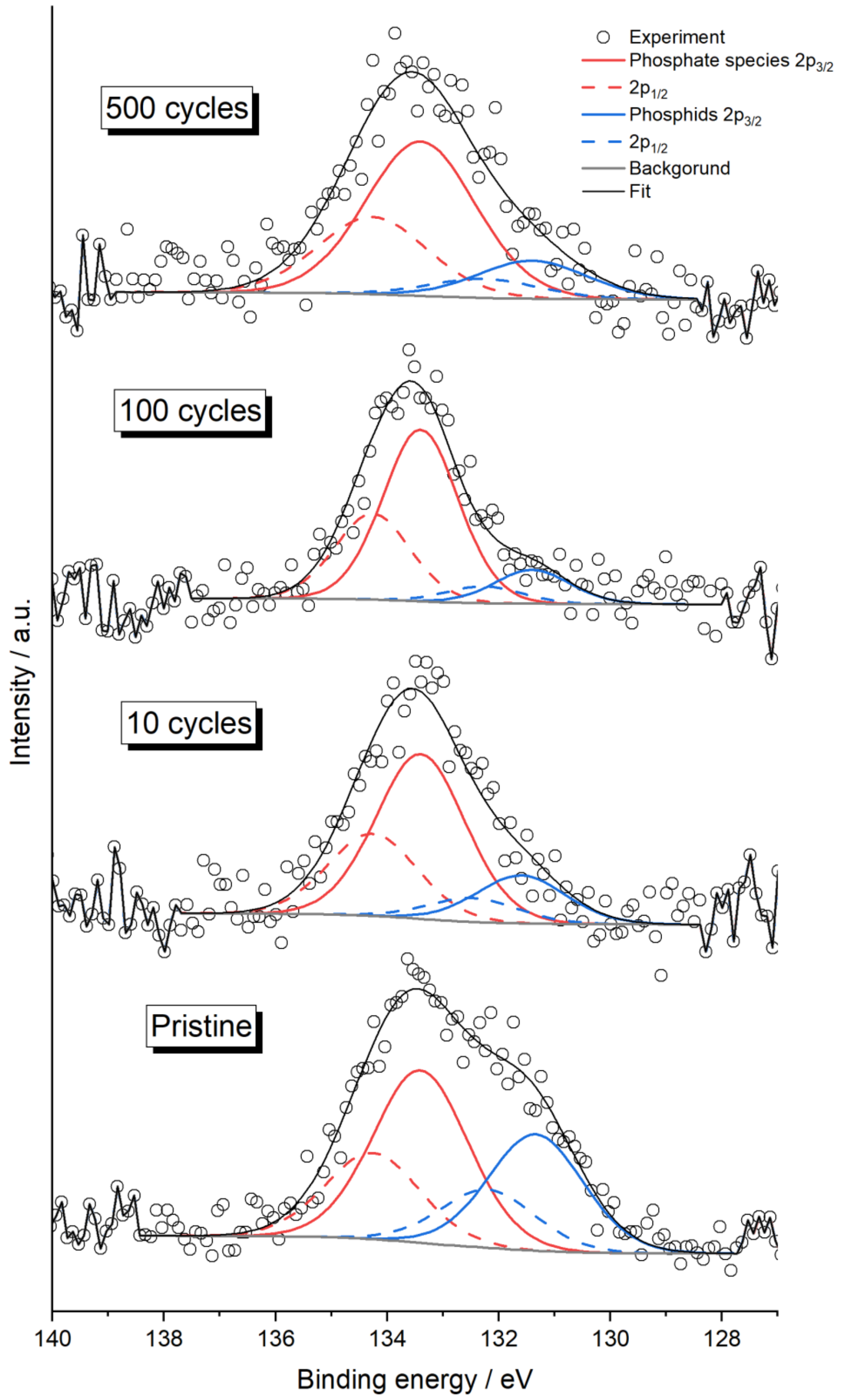

Figure S4. Fitted P 2p XPS spectra of $NiFe_2O_4$-5P in pristine state and after 10 cycles, 100 cycles, and 500 cycles. The red curves correspond to phosphate-like species (~133.4 eV), and the blue curves represent phosphide-like species (~131.4 eV). The deconvolution shows that while the phosphide component diminishes with increasing cycles, the phosphate species remains predominant, consistent with progressive oxidation or dissolution of reduced P under OER conditions.

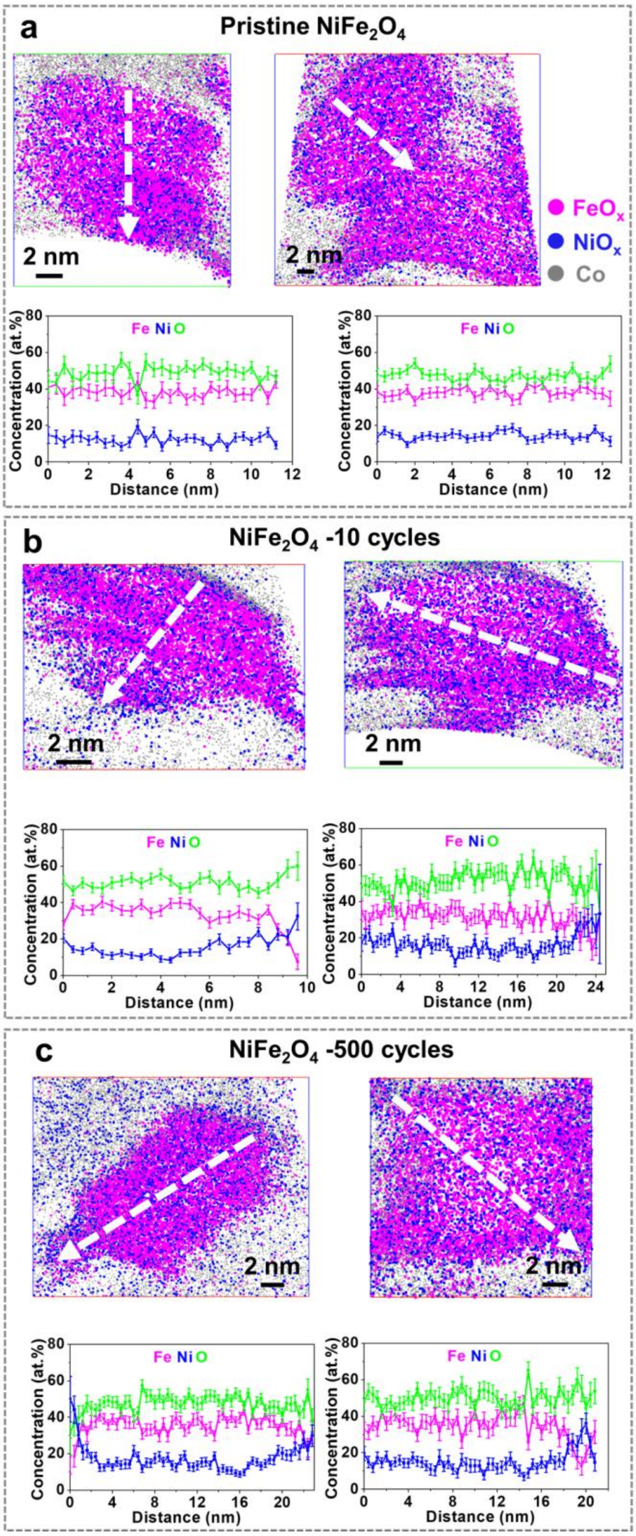

Figure S5. APT analysis of other $NiFe_2O_4$ nanoparticles. Atom maps and 1D concentration profiles of $NiFe_2O_4$-5P nanoparticles (a) at the pristine state, (b) after 10 cycles and (c) after 500 cycles. The error bars of the 1D concentration profiles were calculated using the equation of $\sqrt{\frac{c(100-c)}{N}}$, where c and N are the atomic concentration and the total number of atoms within the fixed bin width profile of 0.4 nm, respectively.

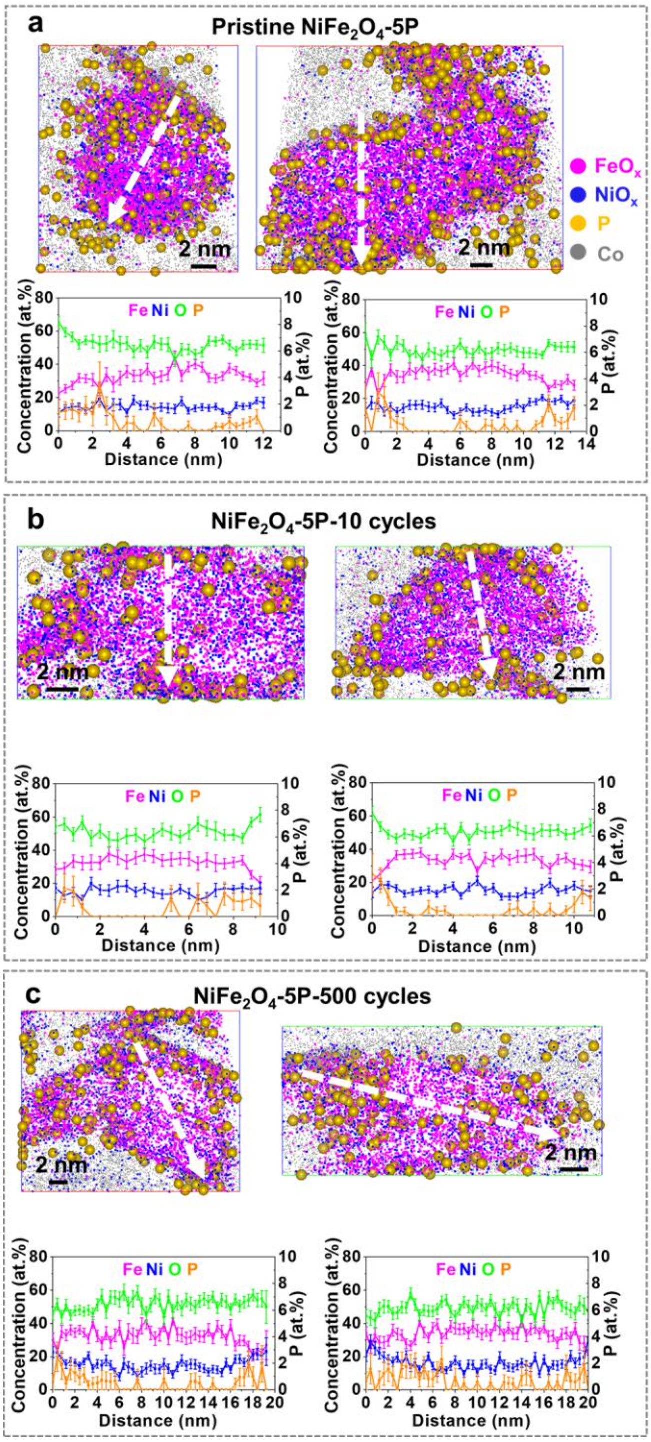

Figure S6. APT analysis of other $NiFe_2O_4$-5P nanoparticles. Atom maps and 1D concentration profiles of $NiFe_2O_4$-5P nanoparticles (a) at the pristine state, (b) after 10 cycles and (c) after 500 cycles. The error bars of the 1D concentration profiles were calculated using the equation of $\sqrt{\frac{c(100-c)}{N}}$, where c and N are the atomic concentration and total number of atoms within the fixed bin width profile of 0.4 nm, respectively.

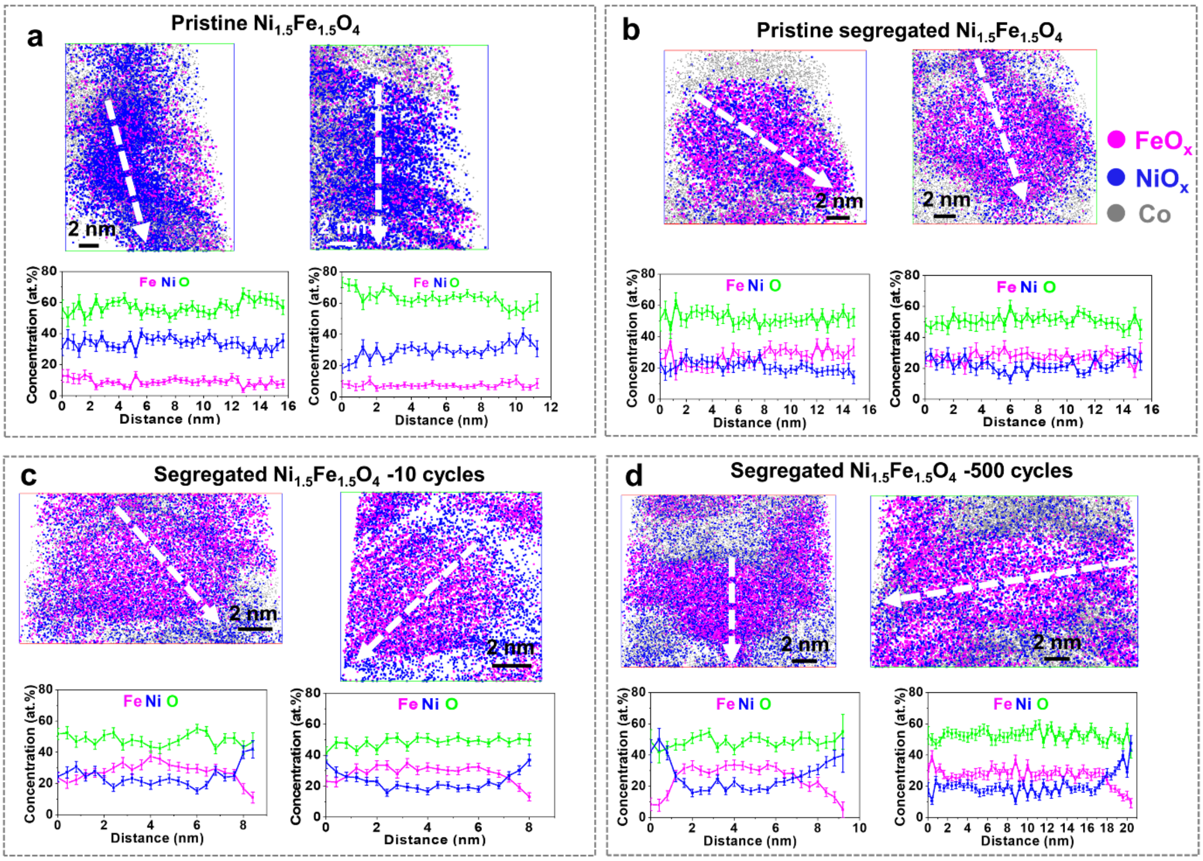

Figure S7. APT analysis of other $Ni_{1.5}Fe_{1.5}O_4$ nanoparticles. Atom maps and 1D concentration profiles of (a) two Ni-rich $Ni_{1.5}Fe_{1.5}O_4$ nanoparticles at the pristine state, and the segregated $Ni_{1.5}Fe_{1.5}O_4$ nanoparticles (b) at the pristine state, (c) after 10 cycles and (d) after 500 cycles. The error bars of the 1D concentration profiles were calculated using the equation of $\sqrt{\frac{c(100-c)}{N}}$, where c and N are the atomic concentration and the total number of atoms within the fixed bin width profile of 0.4 nm, respectively.

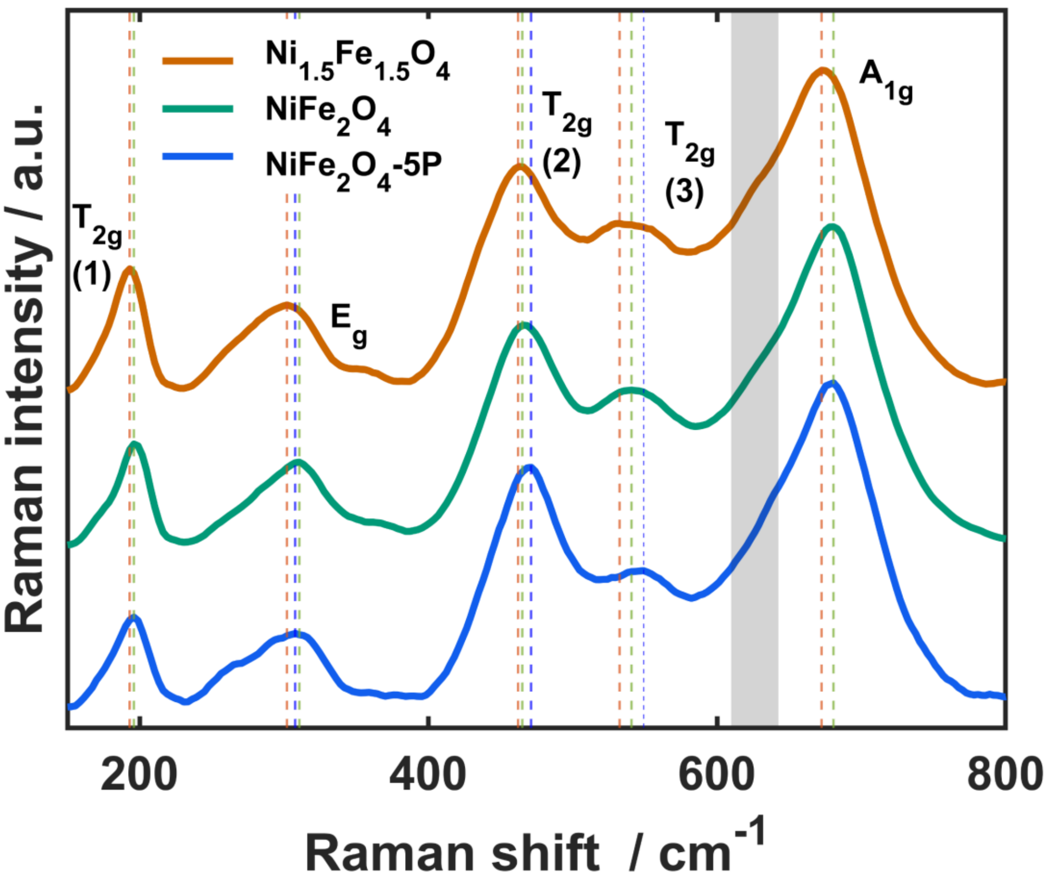

Figure S8. Raman spectra of the different catalysts deposited on a GC electrode. The spectra presented are the mean of five spectra collected at OCP, using 1 s of integration time. Five modes are observed of spinels, i.e., A_{1g}, E_g, three T_{2g}. The peak position and ratios can be used to indicate the structure and octahedral/tetrahedral occupies. Specifically, A_{1g} mode at 670-680 cm^{-1} is related to symmetric stretching of M-O at tetrahedral position. The presence of the shoulder (highlighted by grey box) at around 630 cm^{-1} is assigned to the presence of both cations, i.e., Ni(II) and Fe(III), in octahedral positions, confirming the inverse spinel structure. Note that Raman peaks of pristine $Ni_{1.5}Fe_{1.5}O_4$ have a lower Raman shift, suggesting its mixed spinel structure.

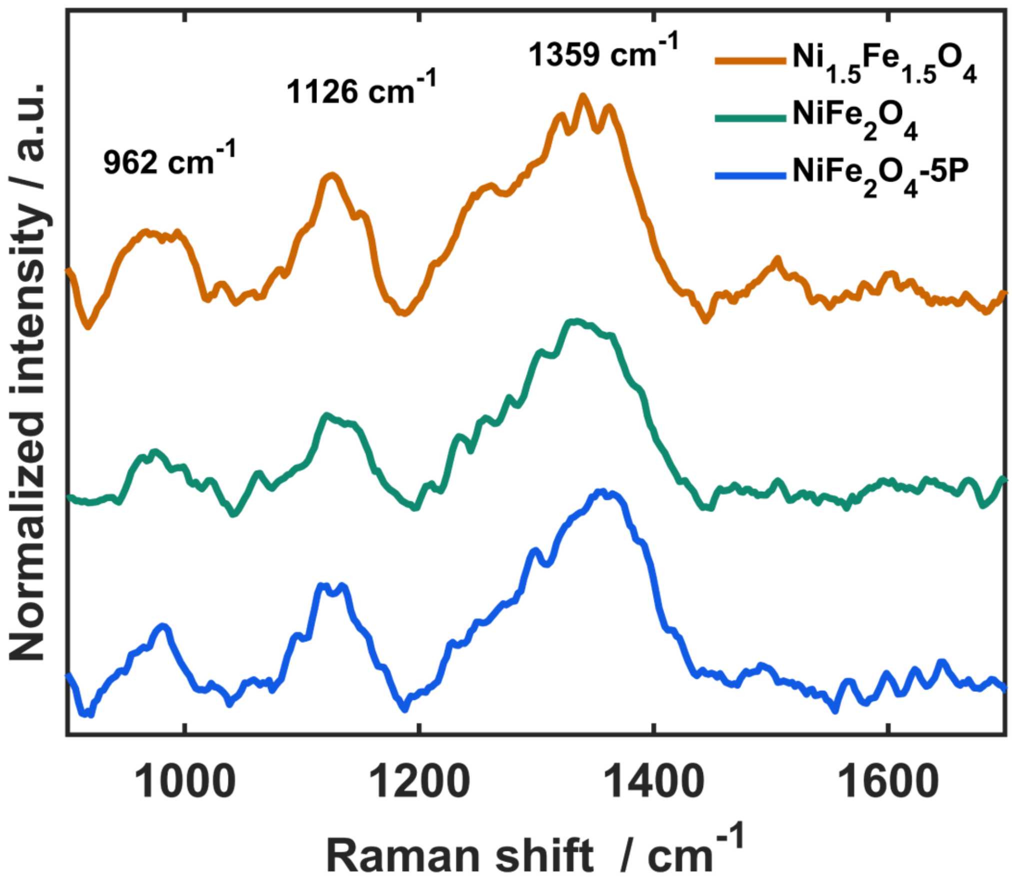

Figure S9. Raman spectra in the region of 900-1700 cm^{-1} for the different catalysts deposited on Au electrodes in 1 M KOH at OCP. The three bands observed in this region are related to the overtones of the T_{2g} (2), T_{2g} (3) and A_{1g} modes[3]. The presence of these bands interferes with the detection of the oxygen active species, which are usually detected between 900 and 1200 cm^{-1}.[4]

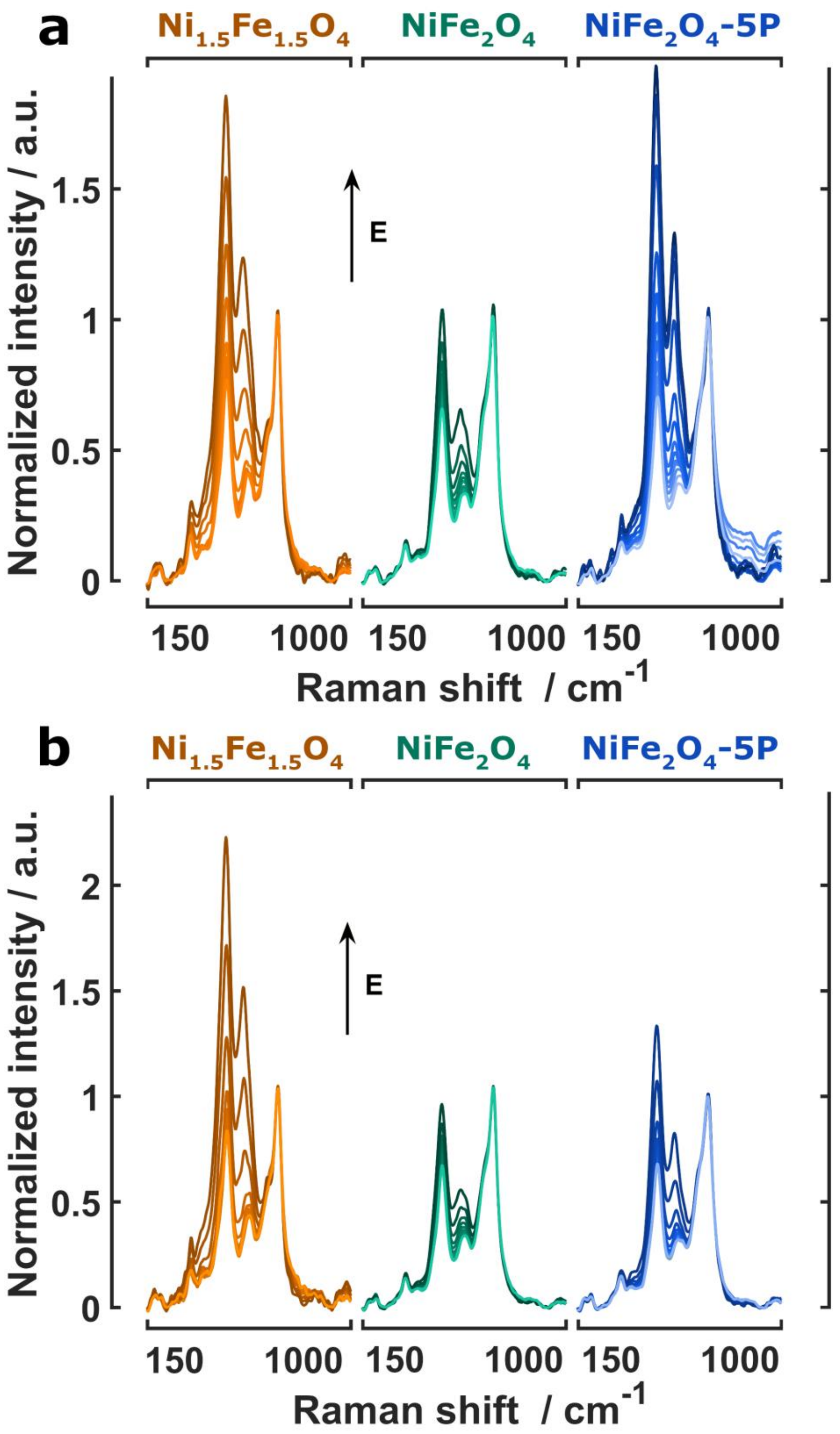

Figure S10. Raman spectra collected during operando surface enhanced Raman spectroscopy measurements during LSV of (a) pristine $NiFe_2O_4$, $NiFe_2O_4$-5P and $Ni_{1.5}Fe_{1.5}O_4$ and (b) 100-cycle $NiFe_2O_4$, 50-cycle $NiFe_2O_4$-5P and 100-cycle $Ni_{1.5}Fe_{1.5}O_4$ in the potential range of 1.1-1.55 V vs. RHE in 1 M KOH.).

References

[1] a) J.-L. Ortiz-Quiñonez , U. Pal , M. S. Villanueva, *ACS omega* **2018**, *3*, 14986-15001; b) V. H. Ong , T. N. Pham , V. M. Tien , N. X. Dinh , N. Thi Lan , N. Van Quy , T. N. Bach , V. D. Lam , L. M. Tung , A.-T. Le, *Journal of Alloys and Compounds* **2023**, *949*, 169880; c) A. Ahlawat , V. Sathe, *Journal of Raman Spectroscopy* **2011**, *42*, 1087-1094.

[2] a) G. Liu , Y. Wu , M. Wang , R. Yao , N. Li , Y. Zhao , F. Zhao , J. Li, *International Journal of Hydrogen Energy* **2019**, *44*, 26992-27000; b) T. J. Frankcombe , Y. Liu, *Chemistry of Materials* **2023**, *35*, 5468-5474; c) D. Zhou , F. Li , Y. Zhao , L. Wang , H. Zou , Y. Shan , J. Fu , Y. Ding , L. Duan , M. Liu , L. Sun , K. Fan, *ACS Catalysis* **2023**, *13*, 4398-4408; d) X. Fan , Y. Ma , A. Sun , X. Zhang , L. Tang , J. Guo, *Surfaces and Interfaces* **2021**, *25*, 101193; e) Z. He , J. Zhang , Z. Gong , H. Lei , D. Zhou , N. Zhang , W. Mai , S. Zhao , Y. Chen, *Nature Communications* **2022**, *13*, 2191.

[3] A. Ahlawat , V. G. Sathe , V. R. Reddy , A. Gupta, *Journal of Magnetism and Magnetic Materials* **2011**, *323*, 2049-2054.

[4] a) B. J. Trześniewski , O. Diaz-Morales , D. A. Vermaas , A. Longo , W. Bras , M. T. Koper , W. A. Smith, *Journal of the American Chemical Society* **2015**, *137*, 15112-15121; b) C. Hu , Y. Hu , C. Fan , L. Yang , Y. Zhang , H. Li , W. Xie, *Angewandte Chemie International Edition* **2021**, *60*, 19774-19778.
